# Functional genomics identifies a small secreted protein that plays a role during the biotrophic to necrotrophic shift in the root rot pathogen *Phytophthora medicaginis*

**DOI:** 10.3389/fpls.2024.1439020

**Published:** 2024-08-19

**Authors:** Donovin W. Coles, Sean L. Bithell, Thomas Jeffries, William S. Cuddy, Jonathan M. Plett

**Affiliations:** ^1^ Hawkesbury Institute for the Environment, Western Sydney University, Richmond, NSW, Australia; ^2^ New South Wales Department of Primary Industries, Tamworth, NSW, Australia; ^3^ School of Science and Health, Western Sydney University, Penrith, NSW, Australia; ^4^ New South Wales Department of Primary Industries, Elizabeth Macarthur Agricultural Institute, Menangle, NSW, Australia

**Keywords:** hemibiotrophic pathogenesis, genome, co-expression network, effector, immune manipulation

## Abstract

**Introduction:**

Hemibiotrophic *Phytophthora* are a group of agriculturally and ecologically important pathogenic oomycetes causing severe decline in plant growth and fitness. The lifestyle of these pathogens consists of an initial biotrophic phase followed by a switch to a necrotrophic phase in the latter stages of infection. Between these two phases is the biotrophic to necrotrophic switch (BNS) phase, the timing and controls of which are not well understood particularly in *Phytophthora* spp. where host resistance has a purely quantitative genetic basis.

**Methods:**

To investigate this we sequenced and annotated the genome of *Phytophthora medicaginis*, causal agent of root rot and substantial yield losses to Fabaceae hosts. We analyzed the transcriptome of *P. medicaginis* across three phases of colonization of a susceptible chickpea host (*Cicer arietinum*) and performed co-regulatory analysis to identify putative small secreted protein (SSP) effectors that influence timing of the BNS in a quantitative pathosystem.

**Results:**

The genome of *P. medicaginis* is ~78 Mb, comparable to *P. fragariae* and *P. rubi* which also cause root rot. Despite this, it encodes the second smallest number of RxLR (arginine-any amino acid-leucine-arginine) containing proteins of currently sequenced *Phytophthora* species. Only quantitative resistance is known in chickpea to *P. medicaginis*, however, we found that many RxLR, Crinkler (CRN), and Nep1-like protein (NLP) proteins and carbohydrate active enzymes (CAZymes) were regulated during infection. Characterization of one of these, *Phytmed_10271*, which encodes an RxLR effector demonstrates that it plays a role in the timing of the BNS phase and root cell death.

**Discussion:**

These findings provide an important framework and resource for understanding the role of pathogenicity factors in purely quantitative *Phytophthora* pathosystems and their implications to the timing of the BNS phase.

## Introduction

Plant pathogens pose a significant threat to agricultural systems and food security worldwide due both to direct and indirect losses in agricultural yields of between 20 and 40% (Savary et al., 2019). To mitigate yield losses in crops, an understanding of how plant pathogens colonize and cause disease in a plant host is required. The genus *Phytophthora* belongs to the oomycetes, a group of filamentous fungus-like eukaryotes that are capable of causing severe economic losses to crops worldwide (Yang et al., 2017). Examples include *Phytophthora infestans*, the cause of late blight in potatoes, and *Phytophthora sojae* the cause of root rot in soybean, together costing >$2 billion US dollars annually in yield losses and control costs (Turner, 2005; Tyler, 2007; Brasier et al., 2022). The genus *Phytophthora* is an major global biosecurity concern due to global plant trade (Scott et al., 2019). Further research is therefore needed into the infection strategies of these economically important plant pathogens to identify new avenues for control. Many species within the genus exhibit a hemibiotrophic lifestyle, i.e., two nutritional modes: nutrients are initially consumed from living plant cells during a biotrophic phase and then a switch to a necrotrophic mode occurs with nutrients obtained from dead cells (Avrova et al., 2008; Zuluaga et al., 2016; Evangelisti et al., 2017; Carella et al., 2018; Coles et al., 2022). In between these two phases of pathogenesis is a “switch” phase, here after referred to as the biotrophic to necrotrophic switch (BNS) phase. Expression profiles during the BNS have been generated and analyzed for *P. sojae* (Moy et al., 2004), *P. infestans* (Zuluaga et al., 2016), *P. capsici* (Jupe et al., 2013) and *P. palmivora* (Evangelisti et al., 2017). These studies have shown the importance of secreted enzymes (e.g., proteases, CAZymes; Palma-Guerrero et al., 2017; Rudd et al., 2015) and secreted pathogenicity factors (e.g. RxLR, CRN, and NLP proteins; O’Connell et al., 2012; Jupe et al., 2013; Rudd et al., 2015). For example, secreted protein *suppression of necrosis 1* (*SNE1*) suppresses programmed cell death mediated by avirulence (AVR)-resistance (R) protein interactions and NLP-induced host cell death in *P. infestans* during early tomato colonization until the transition to necrotrophy (Kelley et al., 2010). However, despite this being a critical phase in the disease progression of hemibiotrophs, the molecular mechanisms regulating transition into and out of this phase are still poorly understood, especially in non-model *Phytophthora* spp. where the genetic basis underlying host resistance is purely quantitative.

Genome sequencing efforts of economically important *Phytophthora* spp. have improved our understanding of how this genus colonizes a susceptible host. Large variation in the size of genomes between *Phytophthora* spp. have been observed. For example, *P. infestans* encodes a relatively large genome (240 Mb) as opposed to *P. sojae* (82 Mb; Tyler et al., 2006). Genomes of other hemibiotrophic *Phytophthora* species including *P. cactorum* (Yang et al., 2018), *P. lateralis* (Quinn et al., 2013), *P. capsici* (Lamour et al., 2012), *P. fragariae* (Adams et al., 2020), and a further 31 recently sequenced *Phytophthora* species have been produced and analyzed, reinforcing the finding that genome size and content vary considerably among *Phytophthora* species (Kronmiller et al., 2023). These studies also revealed that pathogenicity genes encoded by different *Phytophthora* spp. display a large degree of variation and plasticity suggesting the need to study specific host-pathogen interactions (Jiang and Tyler, 2012; Judelson, 2012). Generally, *Phytophthora* genomes are also characterized by a hallmark of”two-speed”genomes: a bipartite architecture whereby one part of the genome is gene rich with minimal repeats and another portion of the genome is characterized by gene sparse, repeat rich regions (Haas et al., 2009; Armitage et al., 2018; Zhang et al., 2019b; Lee et al., 2021; Mandal et al., 2022). Pathogenicity genes such as RxLR effectors often occur within highly repetitive regions of the genome that experience rapid evolutionary change thereby supporting the formation of new virulence mechanisms and enabling evasion of host surveillance and defense systems to promote colonization (Tyler et al., 2006; Haas et al., 2009; Dong et al., 2015; Zhang et al., 2019b). For example, functional analyses show virulent *P. sojae* isolates express the virulent RxLR *AVR3aEM* allele that suppresses host cell death induced by INF1 elicitin while avirulent isolates carry the *AVR3aKI* allele that is recognized by potato R3a resistance protein triggering the hypersensitive response (Bos et al., 2006). Du et al. (2015) showed that avirulent *P. infestans* isolates secrete RxLR effector AVR1 that targets Sec5, a protein involved in host vesicle trafficking but induces the hypersensitive response in resistant potato plants carrying the R1 resistance protein. Virulent isolates lack the *AVR1* locus and instead possess a homolog at another locus termed *AVR1-like*, that is not recognized by potato plants carrying *R1* nor does the virulence factor target Sec5 thereby supporting alternative plant colonization strategies (Du et al., 2015). To date, between 26 and 560 RxLR effectors per genome have been identified in different *Phytophthora* species (Haas et al., 2009; Mandal et al., 2022).

The majority of our knowledge of effectors rests in host models displaying qualitative resistance against different *Phytophthora* species. For *P. infestans*, studies of effectors and associated host resistance genes have provided information on the durability of specific potato varieties in breeding programs (Champouret et al., 2009; Gilroy et al., 2011). Similarly, efforts to understand the genetic diversity and race structure of *P. sojae* have identified resistance genes for soybean breeding programs (Sugimoto et al., 2012). However, we also need to expand our knowledge concerning the role of effectors in *Phytophthora* pathosystems where only quantitative resistance is known. *Phytophthora medicaginis* E.M. Hans. and D.P. Maxwell is an economically important soil- and water-borne hemibiotrophic oomycete pathogen that causes *Phytophthora* root rot (PRR) of lucerne and chickpea (Salam et al., 2011; Schwinghamer et al., 2011; Coles et al., 2022). Chickpeas (*Cicer arietinum* L.) are the second largest cultivated pulse crop in the world after lupin (*Lupinus* L.) and are an increasingly important food source in developed and developing countries (Merga and Haji, 2019). Moderate resistance to *P. medicaginis* has been developed in modern chickpea varieties, resistance that has a quantitative genetic basis whereby the degree of disease is controlled by many genes of small effect (Amalraj et al., 2019). To better understand the molecular aspects of a quantitative *Phytophthora* pathosystem and mechanisms contributing to the BNS in such systems, genomic and transcriptomic resources for *P. medicaginis* during infection of chickpea need to be developed.

In this study, we sequenced the genome of an Australian isolate of *P. medicaginis* and profiled the expression of genes during three phases of hemibiotrophic infection in a susceptible chickpea host to provide an improved understanding of the molecular mechanisms employed during the biotrophic to necrotrophic shift in a quantitative system. We first hypothesized that the *P. medicaginis* genome would contain fewer RxLR effectors compared to other *Phytophthora* species where qualitative resistance mechanisms are present. Secondly, given their role in hemibiotrophic pathogenesis, we hypothesized that *P. medicaginis* would encode SSPs that display phase-specific expression patterns during infection. Lastly, we hypothesized that effector-like SSPs which may play a direct role in influencing plant defense pathways during the BNS phase would co-express with a discrete number of plant defense signaling genes, and that mis-regulation of an SSP meeting these criteria would alter plant colonization. The findings from this study provide a better understanding of effector-like SSPs involved during hemibiotrophic lifestyle switching and their evolution in quantitative systems.

## Materials and methods

### DNA sequencing and assembly

*P. medicaginis* isolate 7831 was baited from soil collected near *Phytophthora* root rot infected chickpea in a commercial crop in Mungallala, Qld, Australia (26.4466° S, 147.5436° E) using the susceptible chickpea var. ‘Sonali’. The pathogenicity of this isolate to chickpea was confirmed by fulfilling Koch’s postulate. For genome sequencing, mycelium of *P. medicaginis* 7831 was grown for five days in liquid V8 medium (containing 200 mL L^-1^ V8™ bottled juice; 3.0 g L^-1^ CaCO_3_) supplemented with ampicillin (100 µg mL^-1^). At harvest, the mycelium was filtered, rinsed and then ground to a fine powder in liquid N_2,_ and DNA extracted according to the method by Kohler et al. (2011). Approximately 7 µg of highly pure DNA was sequenced at GENEWIZ Biological Technology Co., Ltd (GENEWIZ, China) using a PacBio RSII/Sequel SMRT and further refined using Illumina HiSeq/Novaseq 2x150 bp paired end sequencing platform. The diploid genome of *P. medicaginis* was assembled by GENEWIZ Biological Technology Co., Ltd (GENEWIZ, China) with PacBio reads using HGAP4 (version 4.0)/Falcon (version 0.3.0) of WGS-Assembler 8.2 (https://github.com/alekseyzimin/wgs-8). The assembly was corrected using Pilon software with Illumina reads and Quiver (version 1.1.0) with PacBio reads. BUSCO statistics were collected using BUSCO (version 5.7.1; Simão et al., 2015) using the stramenopiles_odb10 database on the *P. medicaginis*, *P. fragariae* BC-16, and *P. sojae* P6497 genome assemblies (Tyler et al., 2006; Adams et al., 2020). Analysis of repeat sequences was performed with Repeatmasker version open-4.0.6 (Smit and Hubley, 2015) and RepeatModeler version 1.0.8 (Smit and Hubley, 2015).

### Gene prediction and functional annotation

Prediction of protein coding genes was performed using Prodigal (version 3.02; Hyatt et al., 2010) and Augustus (version 3.3; Stanke et al., 2006). The protein coding genes were annotated on the National Centre for Biotechnology Information (NCBI) nonredundant database using Diamond BLASTp with an *E* < 0.001 while other arguments were set to default. Gene functions were annotated using the Gene Ontology (GO) database on Blast2GO by BLASTp with an *E* < 0.001 (Gene Ontology Consortium, 2004). Pathways analysis was performed using the Kyoto Encyclopedia of Genes and Genomes (KEGG) database by BLASTn using an *E* < 1.0 x 10^-5^ (https://www.genome.jp/kegg/pathway.html; Kanehisa and Goto, 2000). Protein classification was performed using eukaryote Clusters of Orthologous Groups (KOG) database by rpstblastn with an *E* < 1.0 x 10^-5^ (https://www.ncbi.nlm.nih.gov/research/cog; Tatusov et al., 2003). CAZymes were annotated using diamond BLASTp on the Carbohydrate-Active enZYme (CAZy) database with an *E* < 0.001 (http://www.cazy.org/; Drula et al., 2022). Transporters were annotated by BLAST on the Transporter Classification Database (TCDB) with an *E* < 1.0 x 10^-5^ (https://www.tcdb.org/; Saier et al., 2006). Non-coding RNA including rRNA, tRNA, snRNA, snoRNA and microRNA were predicted using tRNAscan-SE (version 1.3.1; Lowe and Eddy, 1997), RNAmmer (version 1.2, http://www.cbs.dtu.dk/services/RNAmmer/; Lagesen et al., 2007) and mapping Rfam (version 12.2; https://rfam.xfam.org/). Putative secreted proteins were identified in the genome of *P. medicaginis* by identifying proteins with a signal peptide using SignalP4.0 (cut-off probability = 0.8) and lacking a transmembrane domain using TMHMM software (Krogh et al., 2001; Almagro Armenteros et al., 2019). The putative secreted proteins were filtered for amino acid length of less than 300 to obtain a set of small secreted proteins using a python script (Feldman et al., 2020). EffectorP was used to predict effectors in the oomycetes secretome (https://effectorp.csiro.au/; Sperschneider and Dodds, 2022). The effectR package in R (version 4.0.3; Tabima and Grünwald, 2019) was used to predict RxLR and CRN proteins from the proteome of *P. medicaginis* by performing both REGEX and HMM searches (**Data Sheet 2**; Haas et al., 2009). The NLP proteins were identified as above but with the custom motif “GHRHDWE” (**Data Sheet 2**; Feng et al., 2014). Visualization of protein sequence domain structures were performed using DOG (Domain Graph, version 1.0; Ren et al., 2009). High confidence RxLRs and NLPs were selected as those containing an RxLR or ‘GHRHDWE’ motif, respectively, and a secretion signal for further analysis. The signal peptide in oomycete CRNs are not always detected by SignalP, therefore putative CRNs containing LFLAK and HVLV motifs were kept for further analysis (Yin et al., 2017). Protein molecular weight were determined using Protein Molecular Weight (https://www.bioinformatics.org/sms/prot_mw.html).

### Microcosm plant infection assay

Seeds of the *P. medicaginis* susceptible chickpea variety ‘Sonali’ were obtained from the New South Wales Department of Primary Industries (NSW DPI, Tamworth, NSW, Australia). Microcosms of 15-day-old chickpea seedlings and *P. medicaginis* were set up according to the method by Coles et al. (2022). After inoculation, the root zones were covered in aluminum foil and the seedling microcosms were placed back into the growth chamber with 15 h light/9 h dark cycle at 18°C, 70% relative humidity and 200-500 μmol light intensity. Root rot lesions were photographed using a Leica EZ4 stereo microscope (Leica, Australia). Four independent axenic subcultures of *P. medicaginis* (i.e., plant-free control tissues) were prepared by aseptically transferring mycelium coated membranes growing on V8 juice agar plates (containing 200 mL L^-1^ V8™ bottled juice; 3.0 g L^-1^ CaCO_3_, 15.0 g L^-1^ bacteriological agar) supplemented with ampicillin (100 µg mL^-1^) onto modified full strength MS media for 12 hours at 25°C in the dark. Mycelium were harvested and snap frozen in liquid N_2_ and stored at -80°C. Staining and confocal microscopy were performed according to the method by Coles et al. (2022).

### RNA-sequence data processing and gene ontology enrichment analysis of unique gene clusters

RNA-sequencing data from four biological replicates of chickpea roots inoculated with *P. medicaginis* isolate 7831 at the three stages of plant colonization (i.e., biotrophic (12 hpi), BNS (24 hpi), and necrotrophic (72 hpi) phases) were obtained from GEO repository accession GSE182741 (Coles et al., 2022). Each biological replicate consisted of two root segments of 1.5 cm in total length surrounding the inoculated site for each timepoint. RNA was extracted from independent *P. medicaginis* axenic cultures using the Bioline Plant II RNA extraction kit (Bioline, Australia) according to the manufacturer’s guidelines. Poly-A RNA libraries were prepared and sequenced by GENEWIZ (Suzhou, China) using an Illumina HiSeq platform and 150bp paired-end configuration. Quality of raw RNA-seq data was assessed using FASTQC (version 0.10.1). All RNA-sequencing reads were then trimmed to remove low-quality sequences and adapters, and aligned to the primary transcripts of *P. medicaginis* genome and *Cicer arietinum* Desi genome (Jain et al., 2013) using CLC Genomics Workbench 12 (Qiagen, Denmark). For the alignment, the minimum length fraction was set to 0.8, the minimum similarity fraction to 0.9 and the maximum number of hits per read was set to 10. Raw unique read counts were determined for each transcript using CLC Genomics Workbench 12 (Qiagen, Denmark). Raw read counts were filtered to keep transcripts that had at least 10 counts in at least one sample (axenic control and timepoints post inoculation). The Bioconductor package DESeq2 (version 1.26.0; Love et al., 2014) was used to normalize raw transcript counts and identify differentially expressed genes. Statistically significant differentially expressed *P. medicaginis* genes at 12, 24 and 72 hpi were identified compared to axenic control using the Benjamin-Hochberg test for multiple testing with a false discovery rate (FDR) of *p* < 0.05. Only transcripts with an average Log_2_FC > 1 and < -1 compared to axenic control were kept for further analysis. Principal component analysis (PCA) was performed to assess the variability between samples in R (version 4.0.3; R Core Team, 2020). Identification of uniquely regulated genes at each timepoint and gene ontology enrichment were performed according to the method by Coles et al. (2022) and TopGO package version 2.36.0, respectively in R version 4.0.3 (Alexa and Rahnenführer, 2009). Heatmaps of regulated *P. medicaginis* genes at each phase of infection were generated using Morpheus (https://software.broadinstitute.org/morpheus/). Identification of unique and common *P. medicaginis* genes regulated at each phase of infection were determined using Venny version 2.1.0 (https://bioinfogp.cnb.csic.es/tools/venny/).

### Time course reactive oxygen species quantification

Roots of six biological replicates inoculated with *P. medicaginis* were harvested at 0, 12, 24 and 72 hpi, and stained with CM-H2DCFDA (Thermo Fisher Scientific, Australia) for 20 minutes as previously described (Chen et al., 2016; Cai et al., 2017; Mak et al., 2021). Roots were then de-stained for 5 minutes with dH_2_O and the fluorescence captured using a TCS SP5 confocal laser scanning microscope (Leica, Australia). For detection of ROS using fluorescence release as a proxy, an excitation wavelength of 488 nm and an emission wavelength of 505 – 540 nm was used. A 20x magnification was used with the zoom setting set to 1x, and the gain was set to 600. Photographs of six replicates were generated for each timepoint and the settings were kept consistent across timepoints and samples.

### Co-expression network analysis between *P. medicaginis* SSPs and chickpea genes

RNA-sequencing data from sixteen samples, including four biological replicates from each timepoint 12 hpi (biotrophic phase), 24 hpi (BNS phase) and 72 hpi (necrotrophic phase) and control (0 hpi) were used for network construction and analysis. Axenically grown cultures was the control when mapping was performed to the *P. medicaginis* genome, while mock-inoculated plant tissue was the control when mapping was performed to the *Cicer arietinum* Desi genome (Jain et al., 2013), RNA-seq data from mock-inoculated plant tissue consisting of two 1.5 cm root segments with mycelium-free V8 juice agar blocks overlaid onto 0.5 x 0.5 mm^2^ sterile membranes at the center of the roots were used as the control and obtained from GEO repository accession GSE182741, Coles et al. (2022). Raw read counts were filtered and normalized as described above using DESeq2 which normalizes transcripts using the median of ratios method (version 1.26.0; Love et al., 2014). For *P. medicaginis*, normalization was performed using all axenic control and timepoints post inoculation samples (i.e., 12, 24 and 72 hpi) while for chickpea normalization was performed using mock-inoculated roots and 12, 24 and 72 hpi samples. The normalized *P. medicaginis* putative small, secreted protein (SSP) transcripts and chickpea normalized transcripts were combined to generate pairwise relationships and build the co-expression network. Gene relationships were determined using Mictools (https://github.com/minepy/mictools). MICe is a consistent estimator of the MIC (Maximal Coefficient Estimator) population value (MICe) that is part of the Mictools pipeline and is used to rank significant gene association by strength (Albanese et al., 2018). A high cut-off of ≥0.8 MICe used to filter the network was chosen based on the sample size used to construct the network and to select only the strongest gene association for further analyses based on previously published guidelines for network construction (Albanese et al., 2018). The gene associations between the RxLR SSPs common to the biotrophic and BNS phase including *Phytmed_10271*, *Phytmed_14744*, *Phytmed_17143*, *Phytmed_3407*, *Phytmed_8330*, *Phytmed_8971*, and *Phytmed_601*, and their putative host co-expressed genes were filtered using a Linux script to investigate if any of the host genes that the RxLRs might regulate play a role in defense. GO enrichment analyses of the host genes correlated with each of the above mentioned RxLR SSPs was performed using the TopGO version 2.36.0 package in R (version 4.0.3; Alexa and Rahnenführer, 2009; R Core Team, 2020). The gene associations between the *Phytmed_10271* SSP and plant genes were visualized using (Cytoscape version 3.8.0; https://cytoscape.org/release_notes_3_8_0.html; Saito et al., 2012). A mapping file was used to visualize the nodes corresponding to SSP as red nodes and plant genes as grey nodes. The width of edges between nodes was mapped based on the MICe associations between nodes and the strength of the correlations was color coded based on Spearman rank (coded by a color code of yellow to purple representing -1 and +1; Hou et al., 2022). The directionality of the Spearman rank correlations between nodes were mapped with positive correlations as solid lines and negative correlations as dashed lines.

### Knockdown of *Phytmed_10271* expression in *P. medicaginis* during chickpea colonization and RNA-sequencing

We verified that *P. medicaginis* genome contains components of RNA silencing machinery including Argonaute and Dicer genes and previous reports show the successful use of dsRNA to silence endogenous genes in *Phytophthora infestans* (Vetukuri et al., 2011). Double stranded interfering RNA (dsiRNAs) were designed using the gene sequence of *Phytmed_10271*. Two sequences of 27 nucleotides in length, termed 10271_dsiRNA_1 and 10271_dsiRNA_2 (**Supplementary Table S1**), were designed and synthesized at IDT (IDT, Australia). An equimolar cocktail containing two dsiRNAs targeting each gene respectively was prepared at a final concentration of 20 nM in nuclease free DEPC treated ddH2O. For the control treatment, scrambled dsiRNA, 5’- cuuccucucuuucucucccuug-3’, was prepared at a final concentration of 20 nM in sterile water. The scrambled dsiRNA served as a negative control (i.e. unable to silence any genes) as BLASTn of the scrambled sequence to the genomes of *P. medicaginis* and *C. arietinum* generated zero hits in either case, revealing the scrambled control dsiRNA shares no homology to the oomycete and the legume. Two hours after inoculation of chickpea var. ‘Sonali’ roots with *P. medicaginis* isolate 7831 as above, we treated the *P. medicaginis* inoculation site with nebulized dsiRNA mixtures using MAD nasal atomization devices (Teleflex, Australia). This process was repeated every 12 hours to maintain repression of the target genes for the duration of the experiment. Root tissue were harvested at 24 hpi from three replicates each per treatment and snap frozen in liquid nitrogen before freezing at -80°C. Each replicate contained two root pieces of 1.5 cm in length with the infected site at the center of each root piece. RNA sequencing, filtering and mapping were performed according to the method by Coles et al. (2022) and described above. Hierarchical clustering of the significantly differentially regulated chickpea genes were performed using Euclidean distance metric and Average linkage by gene expression on Morpheus (https://software.broadinstitute.org/morpheus/).

### Electrolyte leakage root cell death assay

Electrolyte leakage cell death assay was carried out according to the method by Coles et al. (2022) with some modifications. Briefly, relative electrolyte leakage of 1.5 cm root pieces with *P. medicaginis* at the center was determined for each treatment sprayed with: 1) *Phytmed_10271* dsiRNA, 2) scrambled dsiRNA (control), and 3) sterile ddH_2_O (RNA-negative control). Sampling for each treatment occurred at 24 hours post inoculation (hpi) with five biological replicates per treatment. In addition, to assess the effect of the *Phytmed_10271* and scrambled dsiRNA’s on plant health, relative electrolyte leakage measurements were determined for roots in the absence of pathogen challenge. Sampling was performed at 24 hpi as above with four biological replicates per treatment, except the RNA-negative control for which three biological replicates were used. Statistical analysis was performed on relative electrolyte leakage data using ANOVA with the Tukey method and car package in R (version 4.1.0; Fox et al., 2013; R Core Team, 2021). *Post hoc* comparison among groups was performed using the emmeans package in R (version 4.1.0; Lenth et al., 2018; R Core Team, 2021). Data visualization was performed in R (version 4.1.0) with ggplot2 (Wickham, 2011; R Core Team, 2021).

### Hyphal extension assay

V8 juice agar plates (3.0 g L^-1^ CaCO_3_, 15.0 g L^-1^ bacteriological agar) supplemented with ampicillin (100 mg/mL) were inoculated with agar plugs of 0.5 mm in diameter of *P. medicaginis* isolate 7831 mycelia and placed in a 25°C incubator. The oomycete colonies were sprayed using a MAD nasal atomization device as described above directly after plating (T0) with each treatment: 1) *Phytmed_10271* dsiRNA, 2) scrambled dsiRNA and 3) sterile water (RNA-negative control) and every 10 hours after plating for 24 hours. Radial colony growth measurements were taken at two locations/colony from five replicates per treatment at 2, 12 and 24 hours after the initial dsiRNA spray. In the case of the RNA-negative control four replicates were used. Statistical analysis data visualization were performed using R as above mentioned.

## Results

### The genome of *Phytophthora medicaginis* contains relatively few secreted RxLR effector gene sequences

To better understand the genetic mechanisms underlying *Phytophthora*-plant interactions with only quantitative resistance mechanisms known, we sought to sequence the genome of a *Phytophthora medicaginis* isolate infectious towards chickpea. *P. medicaginis* isolate 7831 infection of chickpea roots lead to the development of brown to black necrotic lesions (**Supplementary Figure S1A**) associated with mass sporulating hyphae (**Supplementary Figure S1B**). *P. medicaginis* has previously been shown to display hemibiotrophic infection in chickpea roots with the presence of a biotrophic, BNS and necrotrophic phase (Coles et al., 2022). Sequencing and assembly of the *P. medicaginis* isolate 7831 reference genome from actively growing mycelium generated a 78 Mb genome in 94 contigs with an N50 value of 1,905 Kbp, slightly smaller than other root rot oomycetes *P. sojae* and *P. fragariae* (**Table 1**). The *P. medicaginis* assembly contained 99 of 100 stramenopiles Benchmarking Universal Single-Copy Orthologs (BUSCO) genes compared to 99 in *P. sojae* (Tyler et al., 2006), suggesting a similar level of genome completeness to the well-studied model *P. sojae* assembly (**Table 1**). 41% of the assembly was identified as repeat rich, a larger value than that of *P. fragariae* and *P. sojae* (**Table 1**; Tyler et al., 2006; Adams et al., 2020). A total of 18,099 protein-coding genes, based on primary transcripts, were predicted in the *P. medicaginis* genome assembly, a smaller number than 37,049 and 26,584 for *P. fragariae* and *P. sojae*, respectively (**Table 1**; Tyler et al., 2006; Adams et al., 2020). KEGG pathways were identified for 3,810 *P. medicaginis* coding genes and of these 2,514 genes were categorized into metabolism, 886 genes to cellular process, 1,226 genes to environmental information processing and 920 genes to genetic information processing (**Supplementary Figure S2A**). KOG predictions categorized 17% of the annotated genes into transport and metabolism of carbohydrates, amino acids, nucleotides, lipids, inorganic ions and coenzymes (**Supplementary Figure S2B**). Furthermore, 269 genes were categorized into secondary metabolite biosynthesis, transport, and catabolism (KOG; **Supplementary Figure S2B**). We identified 1,886 CAZymes in the genome of *P. medicaginis* based on BLASTp to the CAZy database. Of these, 24% were categorized as Carbohydrate-Binding Modules (CBMs), 30.2% as Glycosyl Transferases, 2.5% with Auxiliary Activities, 2% Carbohydrate Esterase’s, 36% to Glycoside Hydrolases (**Supplementary Figure S3**). In total, 8,745 (48%) genes were assigned to GO terms, a similar proportion to that for *P. capsici* of 9,017 genes (45%; Maillot et al., 2022). Of the GO annotations, 585 GO IDs and 5,525 gene were categorized into biological process, 816 GO IDs and 8,077 genes into molecular function, and 195 GO IDs and 1,795 genes were categorized into cellular compartment (**Supplementary Figure S4**). Performing BLAST to the Transporter Classification Database (TCDB), 210 membrane transporter proteins were identified. Using *ab initio* and mapping approaches, we identified a total of 1,896 noncoding RNAs (ncRNAs) of which tRNAs accounted for 87.5%, while 127 rRNAs and 109 other ncRNAs were identified (**Data Sheet 3**). RxLR-dEER effectors, are a major class of secreted proteins in oomycete pathogens that are transferred into host cells to manipulate defenses and facilitate colonization (Yang et al., 2018). RxLR effectors possess a conserved RXLR (arginine-any amino acid-leucine-arginine) motif making it possible to identify them in oomycete genomes (Win et al., 2007). We identified 39 RxLR effectors from the secretome of *P. medicaginis* containing an RxLR-only or RxLR and EER motif (**Table 2**; **Supplementary Table S2**). In addition to RxLR effectors, oomycetes pathogens secrete a large number of Crinkler (CRN) effectors. CRNs are characterized by conserved N-terminal LFLAK motif and recombination site HVLV motif (Haas et al., 2009). Previous research shows that the CRN signal peptide are not always detected by SignalP analysis (Yin et al., 2017). Given this, we identified 56 CRNs from *P. medicaginis* gene models containing both a LFLAK and HVLV motif (**Table 2**; **Supplementary Table S3**). Of these, only eight were predicted to contain a signal peptide based on signalP4.0-HMM (**Table 2**). *Phytopthtora* spp. also secrete Nep1-like protein (NLP) which are toxins that induce necrosis (Qutob et al., 2002). Using the conserved “GHRHDWE” motif, we identified three NLPs with a signal peptide (**Table 2**; **Supplementary Table S4**; Feng et al., 2014).

**Table 1:** Assembly and gene prediction statistics for the *Phytophthora medicaginis* genome compared to *Phytophthora fragariae* (Adams et al., 2020) and *Phytophthora sojae* (Tyler et al., 2006).

	*Phytophthora medicaginis* 7831	*Phytophthora fragariae* BC-16	*Phytophthora sojae* P6497
Assembly			
Assembled genome size (Mb)	78.91	90.97	82.60
Number of contigs	94	180	862
N50 (Kb)	1,905.3	923.5	386.0
Repeat statistics and gene prediction			
Repeatmasked	41%	38%	29%
No. of predicted protein-coding genes	18,099	37,049	26,584
Single-copy BUSCO genes	99 (99%)	99 (99%)	94 (94%)

**Table 2:** Prediction of pathogenicity related proteins in the genome of *Phytophthora medicaginis* isolate 7831.

Category	Putative number
Secreted proteins	991
EffectorP	327
Cytoplasmic effectors	169
Apoplastic effectors	158
RxLR (HMM prediction)	250
RxLR (Regex prediction)	63
Putative RxLR	167
High confidence RxLR	39
CRN (HMM prediction)	2,445*
CRN (Regex prediction)	60
Putative CRN	56
NLP (HMM prediction)	124
NLP (Regex prediction)	9
High confidence NLP	3

EffectorP was performed with *P. medicaginis* secreted proteins. RxLR, arginine-any amino acid-leucine-arginine; CRN, Crinkler and Necrosis; NLP, Nep1-like protein. RxLR, CRN and NLP Regex and HHM searches were performed with *P. medicaginis* proteome (Tabima and Grünwald, 2019). Putative RxLR contain RxLR-only or RxLR and EER motifs (Xu et al., 2024). Putative CRN contain LFLAK and HVLV motifs (Tabima and Grünwald, 2019). *SignalP analysis does not readily detect oomycete CRN signal peptide so only putative CRN are shown here (Yin et al., 2017). High confidence RxLRs and NLP proteins are those that contain an RxLR or ‘GHRHDWE’ motif, respectively, and are predicted to be secreted and lack any transmembrane domain (Feng et al., 2014; Tabima and Grünwald, 2019).

### *P. medicaginis* exhibits phase-specific transcriptomic responses during colonization of chickpea

**Figure 1:**
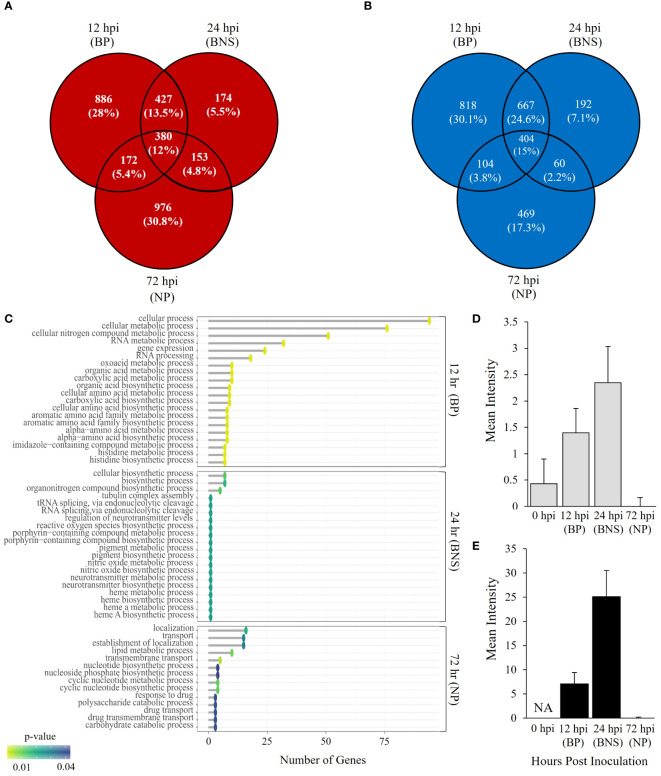
*Phytophthora medicaginis* displays phase-specific Gene Ontology (GO) biological processes during chickpea root infection. **(A)** Venn diagram showing common and unique *P. medicaginis* up-regulated genes at 12-, 24- and 72-hours post inoculation. **(B)** Venn diagram showing common and unique *P. medicaginis* down-regulated gene sets at 12-, 24- and 72-hours post inoculation. **(C)** Enriched GO biological processes associated with combined uniquely up- and down-regulated genes in *P. medicaginis* at the three stages of hemibiotrophic infection. The GO terms are shown on the y-axes. The number of genes associated with each GO term are shown on the x-axes. The Fisher’s exact test p-value statistic for each GO term is shown based on a blue-yellow gradient (*p* < 0.05). **(D)** Mean intensity of reactive oxygen species (ROS) staining in root compartment. **(E)** Mean intensity of ROS staining in hyphae. + SE. The x-axis shows the time points post inoculation in hours and the y-axis shows the mean intensity of ROS staining. BP, biotrophic phase; BNS, biotrophic to necrotrophic switch phase; NP, necrotrophic phase; hpi, hours post inoculation.

We investigated gene expression profiles of *P. medicaginis* in chickpea roots at 12, 24 and 72 hpi, which correspond to the biotrophic, BNS and necrotrophic phases of infection to identify the biological processes governing the BNS of the oomycete (Coles et al., 2022). In total, 5,243 genes, ~27% of predicted genes in the *P. medicaginis* genome, were significantly differentially expressed in at least one of the phases of infection (Log_2_FC > 1 and < -1 and *p* < 0.05, **Supplementary Figures S5A, B**; **Supplementary Table S5**). PCA revealed that 57% of the variance was explained by PC1 separating the samples by early (12 and 24 hpi) and late (72 hpi) stages of infection, while 27% of the variance was explained by PC2 separating the samples by axenic and inoculated treatments (**Supplementary Figure S5C**). 64.3% of significantly up-regulated genes were unique to a timepoint (**Figure 1A**), while 54.5% down-regulated genes were unique to a timepoint (Log_2_FC > 1 and < -1 and *p* < 0.05; **Figure 1B**). Gene ontologies of the uniquely up-regulated genes at 12 hpi were enriched in processes such as histidine biosynthetic process and carboxylic acid biosynthetic process whereas lipid modification, phospholipid dephosphorylation and phosphatidylinositol dephosphorylation were among the enriched processes for the unique down-regulated genes (*p* < 0.05; **Figure 1C**; **Supplementary Table S6**). At 24 hpi, the BNS phase, unique up-regulated genes were associated with heme A biosynthesis, porphyrin-containing compound biosynthesis and nitric oxide biosynthesis while organonitrogen compound biosynthesis and tRNA splicing via endonucleolytic cleavage were among the enriched processes for the unique down-regulated genes (*p* < 0.05; **Figure 1C**; **Supplementary Table S6**). During the necrotrophic phase (72 hpi), uniquely up-regulated genes were associated with cyclic nucleotide metabolic process and drug transmembrane transport while polysaccharide catabolic process, carbohydrate catabolic process and lipid metabolic process were found enriched among the unique down-regulated genes (*p* < 0.05; **Figure 1C**; **Supplementary Table S6**). The identification of ROS biosynthesis as enriched pathway during the BNS led us to investigate ROS further during the three phases of *P. medicaginis* infection. Quantification of ROS revealed that at the BNS ROS was highest in both roots and hyphae compared to the other phases of infection (**Figures 1D, E**).

### *P. medicaginis* genes encoding SSPs display phase-specific regulation

**Figure 2:**
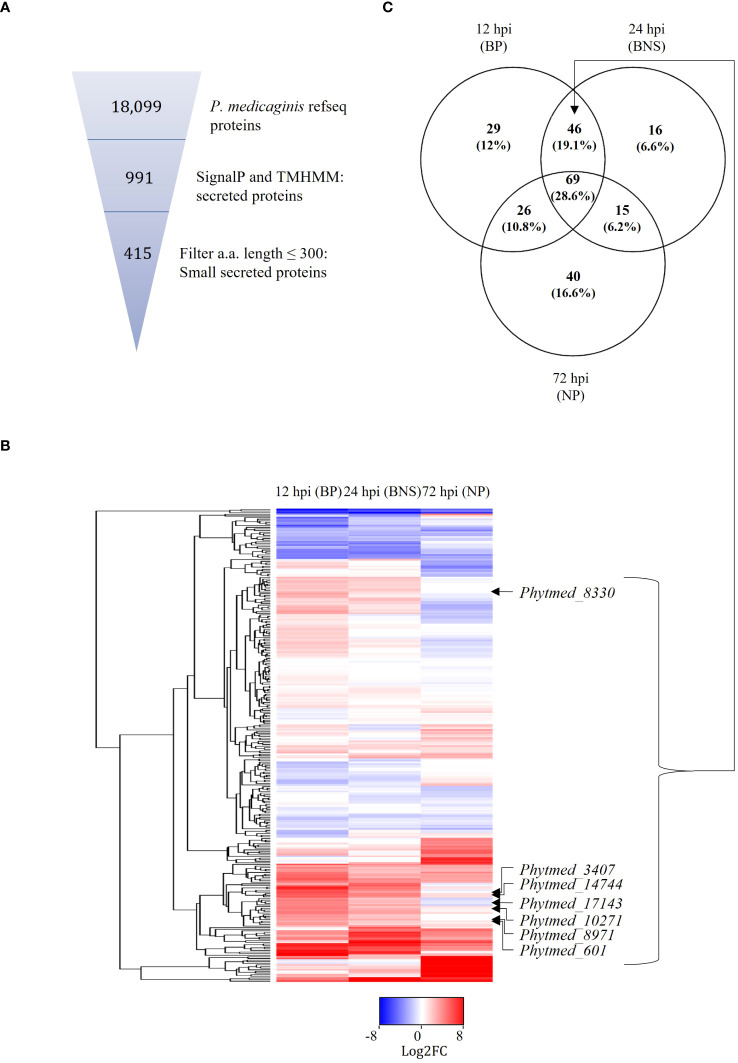
Putative *P. medicaginis* small secreted proteins (SSPs) display phase specific regulation during the lifestyle switch of *P. medicaginis.* **(A)** Identification of SSPs in the genome of *P. medicaginis* using SignalP (Almagro Armenteros et al., 2019) and TMHMM (Krogh et al., 2001) software and filtering for amino acid size < 300 amino acids using a python script. **(B)** Heatmap of 292 SSPs regulated at 12-, 24- and 72-hours post inoculation encompassing the biotrophic, switch and necrotrophic phases, respectively. **(C)** Venn diagram of significantly differentially regulated common and unique SSPs at the three phases of hemibiotrophic infection (*p* < 0.05). BP, biotrophic phase; BNS, biotrophic to necrotrophic switch phase; NP, necrotrophic phase; hpi, hours post inoculation.

Given that SSPs play important roles in the regulation of hemibiotrophic infection (Kim et al., 2016), we sought to annotate these in the genome of *P. medicaginis* and investigate their expression patterns during the biotrophic, BNS and necrotrophic phases in this study. In an effort to annotate these in *P. medicaginis*, we predicted 991 putative secreted proteins from the *P. medicaginis* proteome containing a signal peptide and lacking a transmembrane domain (**Table 2**). The secretome of *P. medicaginis* contained 327 putative effectors and of these, 169 were categorized as cytoplasmic effectors and 158 were categorized as apoplastic effectors (**Table 2**). It is possible that non-canonical open reading frames may contain further effector-like proteins. Filtering of the secreted proteins for a maximum length of 300 amino acids, we obtained a set of 415 SSPs (**Figure 2A**). Of the 415 putative SSP genes, 292 transcripts were detected in *P. medicaginis* during the infection time course in our study (**Figure 2B**; **Supplementary Table S7**) including 29 specific to the biotrophic phase, 16 specific to the BNS phase and 40 specific to the necrotrophic phase (**Figure 2C**). Functional classification of the regulated SSPs revealed putative roles relating to oxidative stress and protein-protein interactions (**Supplementary Table S7**). Ortholog analysis was then performed between 161 P*. medicaginis* SSPs significantly up-regulated during our time course and that of the proteomes of distantly related *Phytophthora* species, *P. palmivora* and *P. infestans*, to identify core SSPs that may regulate each phase across the genus (**Supplementary Figure S6A**). We focused on these three *Phytophthora* species because RNA-seq data at the same three key phases of infection including the biotrophic, BNS and necrotrophic phase was only available for these species. This enabled us to investigate our aim of which core orthologous SSPs are similarly regulated during the same phase of infection in *Phytophthora*. In total, 24 clusters exhibited similar expression patterns in at least two *Phytophthora* species (**Supplementary Figure S6B**). These included cluster 1, pectate lyase, and cluster 22, peptidase, both of which represent core orthologous genes associated to hemibiotrophic colonization in *Phytophthora* (**Supplementary Figure S6B**).

### *P. medicaginis* SSP *Phytmed_10271* co-regulates with chickpea defense signaling pathways

**Figure 3:**
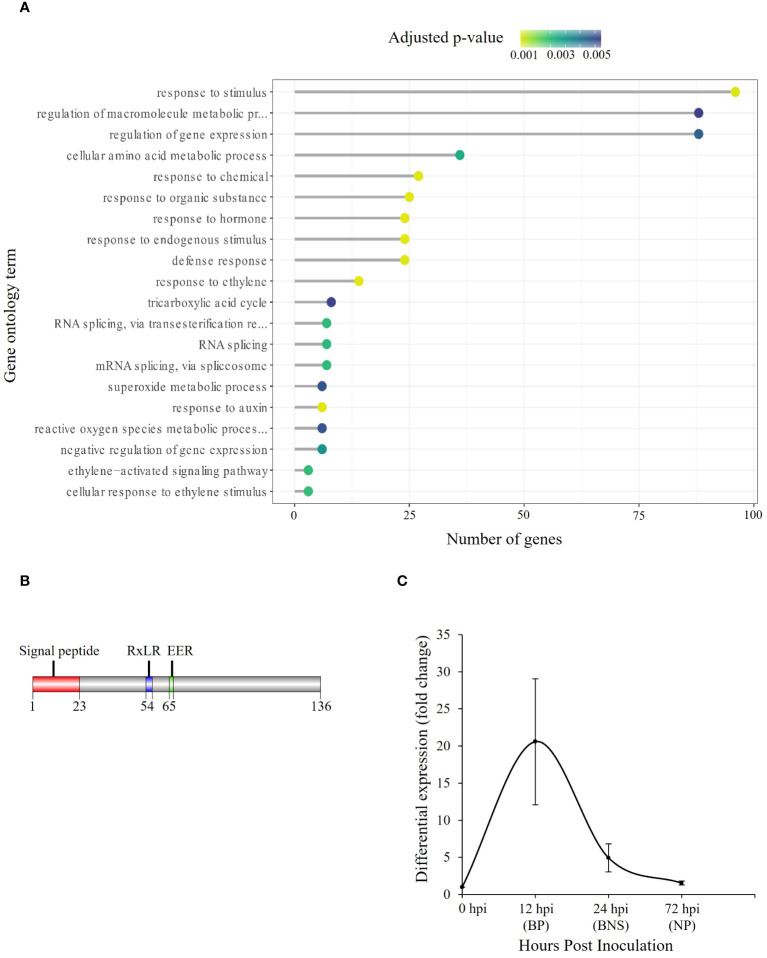
Putative *Phytophthora medicaginis* small secreted protein (SSP) *Phytmed_10271* displays co-regulation with chickpea defense pathways. **(A)** Enriched Gene Ontology biological processes associated with chickpea genes co-expressed with *Phytmed_1027*. The GO terms are shown on the y-axis. The number of genes associated with each GO term are shown on the x-axis. The Fisher’s exact test p-value statistic for each GO term is shown based on a blue-yellow gradient (*p* < 0.05). **(B)** Annotated *Phytmed_10271* protein with motifs present at specific regions within the amino acid sequence. **(C)** Fold change in expression of *Phytmed_10271* relative to control at three phases of hemibiotrophic infection. BP, biotrophic phase; BNS, biotrophic to necrotrophic switch phase; NP, necrotrophic phase; hpi, hours post inoculation. The bars indicate standard error.

Given that RxLRs are known to regulate phases of hemibiotrophic infection (Zuluaga et al., 2016), we were interested in identifying SSPs of this class that were significantly regulated during the BNS phase of *P. medicaginis* to identify RxLRs that could regulate this phase during infection. Of the 415 P*. medicaginis* SSPs, 28 were predicted to be RxLR-like. We did not identify any RxLRs specifically regulated during the BNS phase so we focused on RxLRs induced during both the biotrophic and BNS phases (**Figure 2C**). We identified seven RxLRs significantly induced during the biotrophic and BNS phase, with expression decreasing through the necrotrophic phase including *Phytmed_10271*, *Phytmed_14744*, *Phytmed_17143*, *Phytmed_3407*, *Phytmed_8330*, *Phytmed_8971*, and *Phytmed_601* (**Figures 2B, C**). These RxLRs were investigated further as effectors that may be associated to the control of the transition to necrotrophy. We employed co-regulatory network analysis using gene expression changes across the time course in both the oomycete and the plant to identify the host genes that the RxLRs might influence to regulate infection. In total, the *P. medicaginis* SSPs and host gene co-expression network contained 29,163 nodes (i.e., genes) and 101,197,286 edges (i.e., co-regulatory relationships) of which 58,685,319 edges were positive and 42,511,967 edges were negative (**Supplementary File S1**). Enrichment analysis on the host genes co-expressed with *Phytmed_17143, Phytmed_3407*, *Phytmed_8971*, and *Phytmed_601* revealed that the host genes were primarily involved in biological processes relating to primary metabolism such as peptide and carbohydrate metabolism, and the carboxylic acid cycle (**Supplementary Table S8**). The host genes co-expressed with *Phytmed_8330* were enriched in processes such as ion transport and microtubule-based movement while those host genes co-expressed with *Phytmed_14744* were enriched for chitin metabolism (**Supplementary Table S8**). The host genes co-expressed with *Phytmed_10271* were found to be enriched in pathways involved in plant defense including phytohormones such ethylene and auxin, and ROS metabolism (**Figure 3A**; **Supplementary Table S8**). Given that *Phytmed_10271* was found to be co-expressed with several plant genes linked to immune functions and that one of these pathways included ROS metabolism which we identified as being important during the BNS phase, we sought to characterize this RxLR SSP further (**Figures 1C–E**). Annotation of *Phytmed_10271* revealed the protein has a length of 136 amino acid residues and is cysteine-poor with a putative molecular weight of 15.16 kDa. A signal peptide spans the first 23 residues while RxLR and EER motifs occur at positions 54 and 65, respectively (**Figure 3B**). The putative effector is up-regulated during all three phases of host colonization with highest expression during the biotrophic phase and consecutively decreased expression at subsequent phases (**Figure 3C**). Average normalized transcript counts for *Phytmed_10271*: axenic control = 7.3, 12 hpi = 151.1, 24 hpi = 36.1 and 72 hpi = 11.3. Although *Phytmed_10271* is lowly expressed we still considered it a candidate because the expression level of this gene varies in a manner commensurate to the length of the BP, thus potentially impacting the timing of the BNS and due to work in other models where lowly expressed RxLR effectors such as PexRD8 and Pex36_45-1_ have been shown to be critical to colonization of a host plant (Oh et al., 2009). Phytmed_10271 shares homology with other oomycete proteins including 50% homology to *P. sojae* AVR1b-1 avirulence-like protein (XP_009531296.1) and 46% homology to *P. infestans* putative secreted RxLR effector peptide protein (XP_002998156.1) based on NCBI BLASTp search (**Supplementary Table S9**). Local BLAST analysis to the *P. medicaginis* genome revealed that *Phytmed_10271* shares no homology with other *P. medicaginis* genes.

### *Phytmed_10271* regulates root rot during the BNS phase of infection

**Figure 4:**
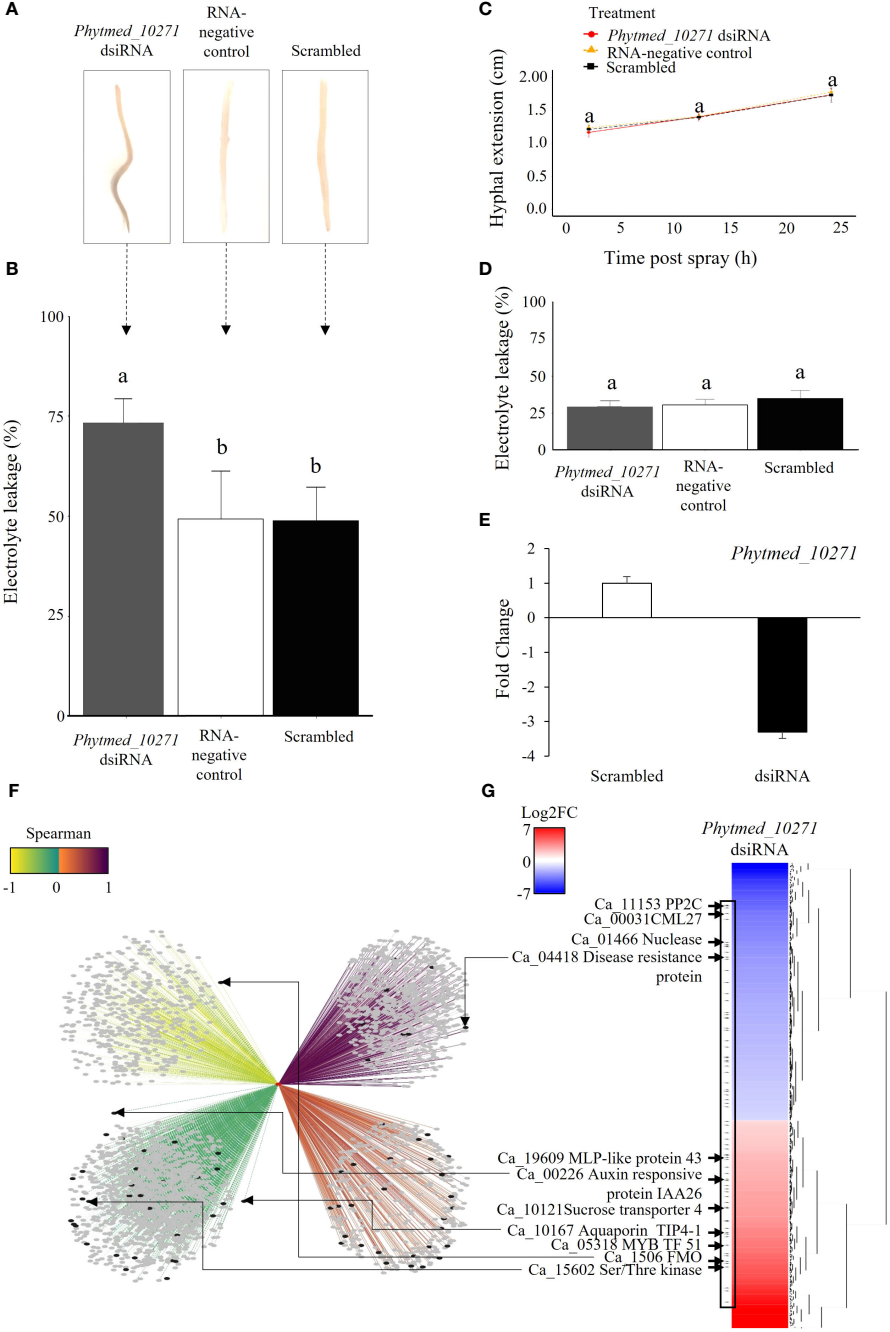
The *Phytmed_10271* knockdown displays a higher rate of tissue necrosis at the biotrophic to necrotrophic switch phase. **(A)** Symptoms in chickpea var. ‘Sonali’ roots, inoculated with *P. medicaginis* and either sprayed with dsiRNA targeting *Phytmed_10271* (Phytmed_10271 dsiRNA), sterile water (RNA-negative control) or scrambled dsiRNA not targeting any gene (control) at 24 hpi, the BNS phase." Here AND is replaced with OR. A representative photo of a root for each treatment with the inoculated site centered in the middle of the root are shown. **(B)** Electrolyte leakage cell death analysis of chickpea var. ‘Sonali’ roots at 24 hpi, the BNS phase. The x-axis shows the treatments including *P. medicaginis* inoculated roots sprayed with *Phytmed_10271* dsiRNA, sterile water and scrambled dsiRNA. The y-axis shows the percentage of electrolyte leakage of pre-boiled relative to post-boiled root samples. Lower case letters indicate significant differences (ANOVA Tukey method). **(C)** Hyphal extension analysis of *Phytmed_10271* dsiRNA, RNA-negative control and scrambled dsiRNA treatments (ANOVA and Tukey *post-hoc* test). **(D)** Electrolyte leakage analysis on roots from plant only set up sprayed with either dsiRNA targeting *Phytmed_*10271, scrambled dsiRNA or sterile water (ANOVA and Tukey test). **(E)** *Phytmed_10271* displays 3x less abundance in the *Phytmed_10271* dsiRNA knockdown treatment compared to scrambled dsiRNA. Data shown are fold change of normalized unique counts in *Phytmed_10271* dsiRNA relative to the scrambled dsiRNA. **(F)** The *Phytmed_10271* SSP and chickpea gene co-expression network. The red node indicates the *Phytmed_10271* SSP, grey nodes indicate chickpea genes, and black nodes indicate the 73 significantly differentially regulated chickpea genes in the *Phytmed_10271* dsiRNA knockdown relative to the scrambled dsiRNA control (Log_2_FC > 1 and < -1, *p* < 0.05). The strength of the edges connecting nodes are shown by a color gradient (Spearman rank: -1 to 1 as yellow to purple). Dotted lines represent negative correlations and solid lines represent positive correlations. **(G)** Hierarchical clustering of the significantly differentially regulated chickpea genes in the *Phytmed_10271* dsiRNA knockdown relative to the scrambled dsiRNA control (Log_2_FC > 1 and < -1, *p* < 0.05). Examples of genes present within the network that were significantly differentially regulated by knockdown of *Phytmed_10271* are highlighted by arrows including IAA26, NDOLE-3-ACETIC ACID INDUCIBLE 26 Protein Phosphatase 2C; CML27, Calcium-binding protein 27; MLP-like 43, Major Latex Protein-like 43; TIP, Tonoplast Intrinsic Protein and FMO, Flavin-binding monooxygenase family protein. BNS, biotrophic to necrotrophic switch; hpi, hours post inoculation; SSP, small secreted protein.

To investigate the role of *Phytmed_10271* during chickpea root rot development, we employed a double stranded interfering RNA (dsiRNA) spray approach for RNA mediated knockdown of the RxLR in a *P. medicaginis-*chickpea microcosm setup (Plett et al., 2019, 2020). Compared to the RNA-negative control (sterile ddH_2_O) and scrambled dsiRNA negative control, the *Phytmed_10271* RNAi knockdown displayed increased root rot browning at 24 hpi, the BNS phase of *P. medicaginis* (**Figure 4A**). Corroborating the disease phenotypic results, repression of *Phytmed_10271* led to significantly higher electrolyte leakage, an indicator of cell death due to rupturing of plasma membranes by the pathogen compared to controls (ANOVA Tukey method, *F* = 12.49, *p* < 0.001; *Phytmed_10271* dsiRNA - RNA-negative control *p* < 0.002, *Phytmed_10271* dsiRNA - scrambled dsiRNA: *p* < 0.002, RNA-negative control - scrambled dsiRNA: *p* = 0.99; **Figure 4B**). The *Phytmed_10271* dsiRNA and scrambled dsiRNA sprays did not affect hyphal extension under axenic conditions (ANOVA Tukey method, *F* = 0.086, *p* ≥ 0.05; **Figure 4C**), nor did the dsiRNA sprays affect plant health in the absence of the oomycete at 24 hpi (ANOVA Tukey method, *F* = 1.48, *p* > 0.05; **Figure 4D**). The dsiRNA spray targeting *Phytmed_10271* led to an average of 3x reduction in transcript expression compared to the scrambled dsiRNA control (T-test, *p* = 0.03; **Figure 4E**). *Phytmed_10271* was co-expressed with 2,731 chickpea genes; 996 were positively co-expressed (solid edges) and 1,735 were negatively co-expressed (dotted edges; **Figure 4F**; **Supplementary Table S10**). All the edges between nodes possess a MICe (Maximal Information Coefficient estimator) > 0.8 as this is a stringent cut-off for networks constructed from the sample size used (Albanese et al., 2018). We then ranked the correlations between *Phytmed_10271* and the host genes by the Spearman rank correlation coefficient to identify the strongest correlations. Of these, 648 host genes displayed a strong positive correlation (dark purple), followed by 348 host genes that displayed a weak positive correlation (orange; **Figure 4F**; **Supplementary Table S10**). For the negatively corelated genes, 462 host genes displayed the strongest correlation for this category (yellow) while 1,273 genes displayed the weakest negative correlation with *Phytmed_10271* (green; **Figure 4F**; **Supplementary Table S10**). We then sequenced the transcriptome of three biological replicates each, the *Phytmed_10271* knockdown and scrambled control, respectively, to validate the network predictions and identify host genes that were actually perturbed by altered expression of the RxLR SSP. In total, we identified 663 chickpea genes that were significantly regulated in the knockdown treatment compared to scrambled control (*p* < 0.05, Log_2_FC > 1 and < -1, **Figure 4G**; **Supplementary Table S11**). Of these, 73 host genes displayed opposite expression patterns with *Phytmed_10271*, in a manner consistent with the Spearman rank predictions of the co-expression network suggesting that these were either directly or indirectly targeted by the activity of the RxLR SSP (black nodes, **Figure 4F**; **Supplementary Table S11**). These included for example a Flavin-binding monooxygenase family protein, Auxin responsive protein IAA26, Serine/Threonine Kinases, Disease Resistance Protein, and aquaporin Tonoplast Intrinsic Protein (TIP) 4; (**Figures 4F, G**; **Supplementary Tables S10**, **11**).

## Discussion

Compared to hemibiotrophic interactions where qualitative resistance has been observed, much less is known about the role of effectors during the BNS in hemibiotrophic pathogens where only quantitative host resistance has been observed. To investigate this, we sequenced the genome and transcriptome of the oomycete, *P. medicaginis* during three phases of hemibiotrophy in chickpea roots. We confirmed our first hypothesis that *P. medicaginis* contains relatively few RxLRs. We also found that *P. medicaginis* RxLR, CRNs and NLPs displayed phase-specific regulation, thereby confirming our second hypothesis. Additionally, we found RxLR-like SSPs co-expressed with plant defense pathways in chickpea, one of which played a role during infection at the BNS phase. The findings from this study provide a better understanding of disease progression in agronomically important quantitative pathosystems by giving evidence that SSPs also have a role in mediating the BNS in such systems as has been observed in qualitative resistance pathosystems.

Of the currently sequenced *Phytophthora* sp. Genomes, *P. medicaginis* genome size and number of coding genes is comparable to other *Phytophthora* species causing root rot such as *P. fragariae, P. sojae*, *P. nicotianae* and *P. rubi* (McGowan and Fitzpatrick, 2020; Mandal et al., 2022). The *P. medicaginis* genome displays the second highest proportion of repeats of the currently published *Phytophthora* genomes after *P. infestans* (Haas et al., 2009). We found that *P. medicaginis* ranks among the *Phytophthora* genomes with low numbers of encoded RxLR effectors, coming second least after *Phytophthora pinifolia* followed by *Phytophthora chlamydospora* and *Phytophthora syringae* (Mandal et al., 2022). Similar to the *P. medicaginis*: chickpea pathosystem, only moderate resistance is present in *Pinus radiata* to *P. pinifolia* (Durán et al., 2010; Ahumada et al., 2013). In other *Phytophthora* spp. such as *P. sojae* and *P. infestans* where qualitative host resistance is present a greater number of RxLR effectors have been Identified, i.e. 350 and 563 for *P. sojae* and *P. infestans*, respectively (Tyler et al., 2006; Champouret et al., 2009; Haas et al., 2009; Sugimoto et al., 2012). The high number of RxLR effectors and their large diversity have been attributed to host selection pressures in qualitative resistance breeding programs between pathogen and host, a theory referred to as the host-pathogen evolutionary arms race (Win et al., 2007; Haas et al., 2009). Compared to *P. infestans* and *P. sojae*, host selection pressure towards *P. medicaginis* is likely to be lower given the relative number of RXLR effectors (Amalraj et al., 2019; Bithell et al., 2023).

Our work characterizing the *P. medicaginis* genome and transcriptome has advanced our understanding of the factors that may contribute the phase switch of this pathogen, and specifically genes associated to the entry and exit of the BNS. PCA analysis of the BNS, BP and NP samples revealed close clustering of the BNS and BP samples suggesting that the gene expression levels between these two phases are relatively similar, although there were still 1,704 differentially regulated genes unique to the BP and 366 differentially regulated genes unique to the BNS. A similar observation has been observed by Jupe et al. (2013) investigating the *P. capsica*-tomato interaction indicating a relatively restricted set of specific molecular pathways regulate the transition between these two phases. We were successful in identifying the unique gene clusters separating the BP and BNS phase samples to pinpoint the metabolic pathways promoting this transition during infection. Of these, ROS and nitric oxide (NO) biosynthetic pathways were identified as enriched signaling pathways during the beginning of the BNS phase. Elevated ROS production has been observed just before the onset of necrotrophy in *Septoria tritici* and *P. parasitica* (Shetty et al., 2007; Wi et al., 2012). Heme A biosynthesis was observed as an enriched response pathway during the *P. medicaginis* BNS phase. Heme is an important component of peroxidase for the detoxification of ROS in *P. nicotianae* during infection of tobacco (Blackman and Hardham, 2008). It is conceivable that high ROS production coupled with efficient antioxidant systems in *P. medicaginis* contribute to the switch to necrotrophy (Jindřichová et al., 2011). A number of CAZymes were also regulated during the BNS phase of *P. medicaginis*, earlier than expectations based on model hemibiotrophs *P. infestans* and *L. maculans* where CAZymes are induced during the late necrotrophic phases of infection (Lowe et al., 2014; Zuluaga et al., 2016). Notably, the genome of *P. medicaginis* contains the highest number of CAZymes compared to other *Phytophthora* spp. sequenced to date (Brouwer et al., 2014; Yang et al., 2018). Of the CAZymes regulated in our study, pectate lyase and glucan endo-1,3-beta-glucosidase were present. Pectate lyases are induced during late phases of infection in hemibiotrophs such as *Coletotrichum* spp. and function to degrade plant tissue to access nutrients (Cnossen-Fassoni et al., 2013; Fu et al., 2015). Likewise, endo-1,3-beta-glucosidase display similar functions in degrading plant cell walls during late phases of infection in hemibiotrophic pathogens such as those in the dothidiomycetes (McLeod et al., 2003; Ökmen et al., 2019). In addition, xylanase encoding SSPs were present also among the CAZymes during the BNS phase. Xylanases have been reported to contribute to the necrotrophic phase of infection in hemibiotrophs such as fungus *Mycosphaerella graminicola* (Siah et al., 2010). In concert with these, necrosis inducing NLP-like encoding SSPs were regulated during the BNS phase of *P. medicaginis*. *P. medicaginis* encodes a similar number of these SSPs as *P. pinifolia* (Kronmiller et al., 2023). NLPs are conserved necrosis inducing proteins across different kingdoms that actively induce plant cell death to facilitate the necrotrophic phase such as has been observed in root infecting *Verticillium dahliae* and *P. sojae* (Qutob et al., 2002; Zhou et al., 2012). These and the CAZymes identified here are likely to form the foundation to facilitate the exit from the BNS phase and the onset of the necrotrophic phase in *P. medicaginis.*

Currently, knowledge of effectors and signaling pathways regulating the BNS phase in non-model quantitative systems is limited. To supplement this gap in our understanding, we specifically focused on this phase of infection in the quantitative *P. medicaginis*:chickpea pathosystem. We identified that 41% of the *P. medicaginis* secretome are predicted to be SSPs. In other hemibiotrophic pathogens, 40-60% of the secretome are predicted to be SSPs suggesting a comparable number in *P. medicaginis* to other hemibiotrophs (Feldman et al., 2020). The fact that chickpea does not respond to *P. medicaginis* in an R gene/AVR qualitative manner (Amalraj et al., 2019), leads to the question concerning the role of SSPs during infection. The majority of the differentially regulated SSPs had no known homologue and/or exhibited the characteristics of effector proteins. For example, SSPs containing RxLR domains often identified as AVR proteins in qualitative pathosystems (Bos et al., 2006), were induced in *P. medicaginis* during infection. It is possible that these sequences have additional roles outside of AVR activity as there are a number of examples where effector-like SSPs have been identified in pathogens whose hosts display quantitative resistance. For example, SSPs regulated during pathogenesis have been identified in *B. collo-cygni* and *Fusarium graminearum* where only quantitative resistance has been observed in the hosts barley and wheat, respectively (Lu and Edwards, 2016; McGrann et al., 2016). Together with our results, these findings would support a significant role of SSPs during infection in quantitative pathosystems.

Identifying pathways regulated by effector-like SSPs where no known R-protein has been isolated can be difficult. Therefore, development of *in silico* analyses such as co-expression networks to narrow the putative pathway(s) impacted by a given SSP would be helpful in advancing this field of research. Through application of co-expression network analysis, we identified a SSP RxLR effector named *Phytmed_10271* that was co-expressed at the transcriptional level with host phytohormone signaling pathways, ROS metabolism, and defense signaling. Similar to our study, Musungu et al. (2016) used co-expression network analysis to show that *Aspergillus flavus* pathogenicity genes were co-expressed with maize ROS cluster during early infection. Likewise, Zhang et al. (2019a) identified *Botrytis cinerea* pathogenicity genes linked to jasmonic-acid and salicylic acid signaling in *Arabidopsis*. In our study, a knockdown of *Phytmed_10271* using dsiRNA during *P. medicaginis* infection of chickpea roots caused increased root cell death during the BNS phase and identification of 73 significantly differentially expressed genes predicted to co-regulate with *Phytmed_10271* in a manner modelled by the co-expression network, thereby confirming the validity of the network topology method as a means of predicting the most probable target pathways of a SSP. Of the differentially expressed genes, Auxin response protein AUX/IAA26 was present and up-regulated in the *Phytmed_10271* knockdown. Previous work by Padmanabhan et al. (2005) showed that a tobacco mosaic virus replicase interacted with *Arabidopsis* AUX/IAA26 to prevent localization of AUX/IAA26 to the nucleus and promoted disease development. Additionally, we found aquaporin TIP4;1 was up-regulated in the *Phytmed_10271* knockdown treatment, a gene associated to lesion formation, vesicle transport and ROS (Ma et al., 2004). These latter pathways were also present within our co-expression network and significantly differentially expressed genes for the SSP knockdown treatment suggesting possible mechanisms for the early root cell death phenotype observed in our study.

## Conclusion

These findings highlight that, while chickpea displays quantitative resistance to *P. medicaginis*, RxLR effectors may still play an important role during the phases of hemibiotrophic infection as observed in qualitative resistance models and the host defense pathways identified in this study may be targeted in future for crop improvement.

## Data Availability

The RNA sequencing datasets generated for this study can be found at the GEO repository with the identifier GSE182741 (https://www.ncbi.nlm.nih.gov/geo/query/acc.cgi?acc=GSE182741) and GSE183604 (https://www.ncbi.nlm.nih.gov/geo/query/acc.cgi?acc=GSE183604. Processed data have been deposited in rds Western Sydney University (https://doi.org/10.26183/mzre-vk16). Data analysis scripts used in this study may be found in rds Western Sydney University (https://doi.org/10.26183/mzre-vk16) and https://github.com/Coles-DW/Phytophthora-medicaginis-differential-expression-analysis. Due to the large size of **Supplementary File S1**, we have deposited the file at https://doi.org/10.26183/mzre-vk16. The raw genome sequencing data have been deposited in the NCBI SRA as part of BioProject PRJNA756124 with accession codes SRX25012627-SRX25012628. The Phytophthora medicaginis isolate 7831 genome assembly and annotation files may be found at rds Western Sydney University (https://doi.org/10.26183/mzre-vk16).
