# Supplementary material for: Functional genomics identifies a small secreted protein that plays a role during the biotrophic to necrotrophic shift in the root rot pathogen Phytophthora medicaginis
Source: Front Plant Sci. 2024 Aug 19;15:1439020. doi: 10.3389/fpls.2024.1439020 (PMC11366588; doi:10.3389/fpls.2024.1439020)
Supplement: Supplementary file 11 [file DataSheet1.docx]

Supplementary Material

Functional genomics identifies a small secreted protein that plays a role during the biotrophic to necrotrophic shift in the root rot pathogen *Phytophthora medicaginis*

**Donovin W. Coles, Sean L. Bithell, Thomas Jeffries, William S. Cuddy, Jonathan M. Plett***

*** Correspondence:** Jonathan M. Plett: j.plett@westernsydney.edu.au

# Supplementary Data

**Supplementary file 1**. R script used to predict RxLR: arginine-any amino acid-leucine-arginine effector, CRN: Crinkler and Necrosis, NLP: Nep1-like protein genes in the genome of *Phytophthora medicaginis* isolate 7831: <https://doi.org/10.26183/mzre-vk16>.

**Supplementary File 2**. *Phytophthora medicaginis* predicted non-coding RNAs.

**Supplementary File 3**. MicE strength and Spearman rank correlations between *Phytophthora medicaginis* small secreted proteins and chickpea genes: <https://doi.org/10.26183/mzre-vk16>.

# Supplementary Figures and Tables

## Supplementary Figures


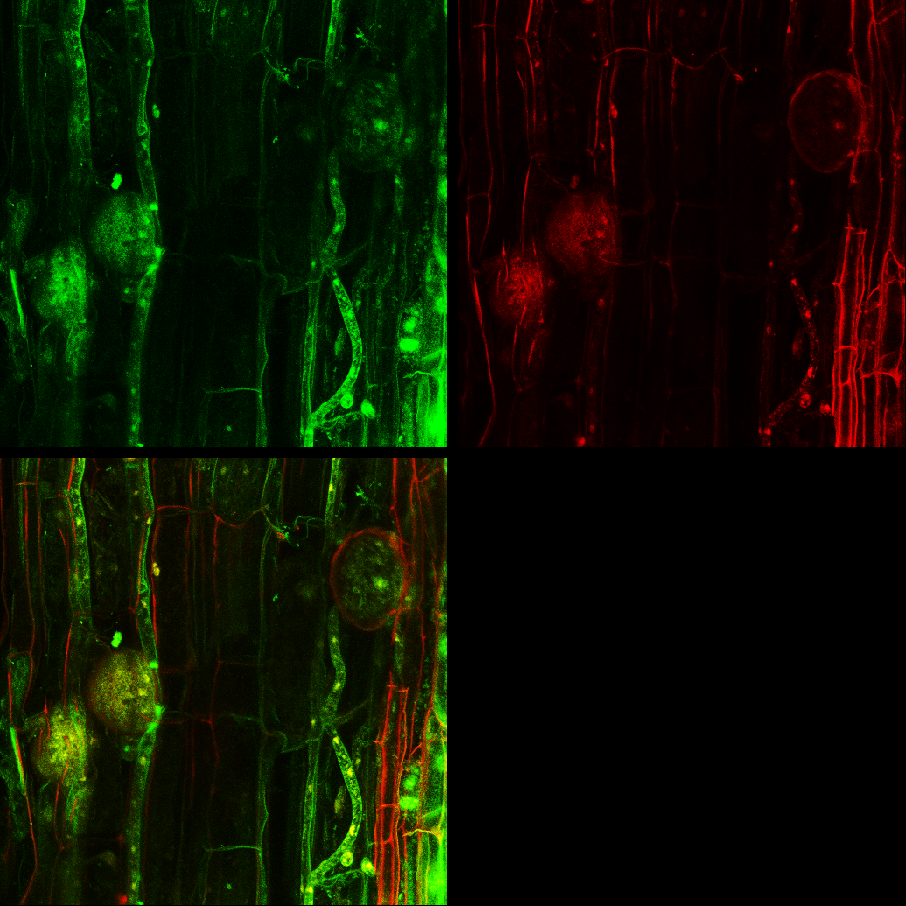


**A**

**B**


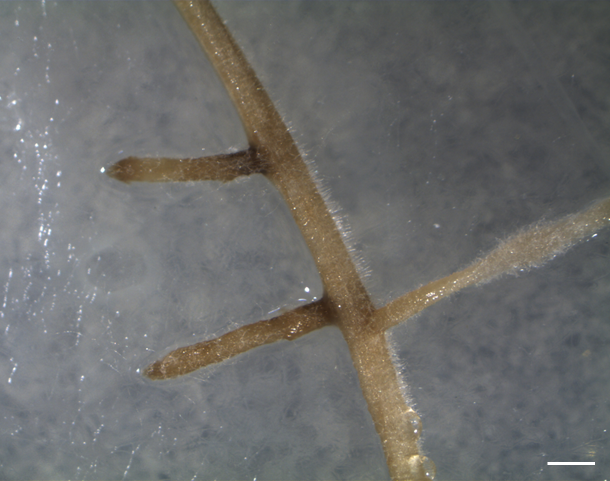


**Supplementary Figure 1.** ***Phytophthora medicaginis* infection of chickpea is associated with root rot and sporulation**. **(A)** *Phytophthora* root rot at the junction between tap root and lateral roots of the susceptible chickpea var. ‘Sonali’ seedling are characterised by necrotic brown/black lesions (black arrows). Scale bar: 1 cm. **(B)** Confocal microscopy view of *P. medicaginis* chlamydospore (green, white arrow) developing within chickpea root cells (red) at the root junction. Scale bar: 50 µm. The image was taken with a longitudinal section of 30 µm in thickness using a TCS SP5 confocal laser scanning microscope.

**A**


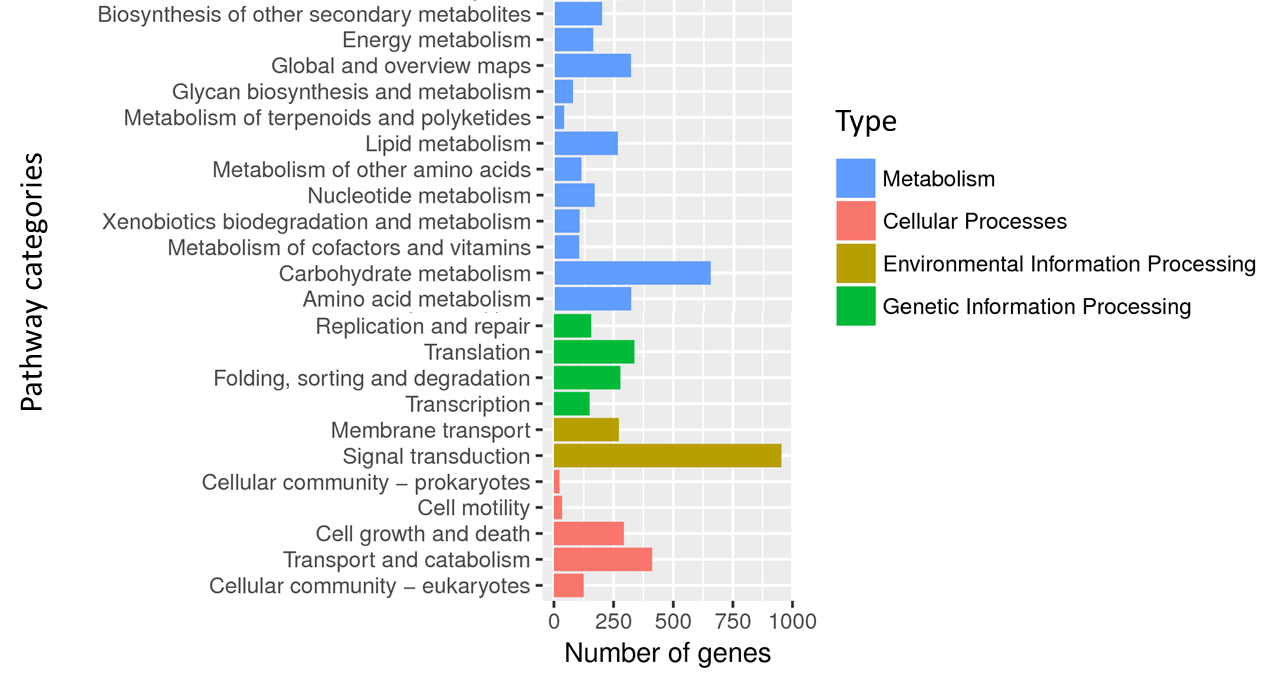


Type

0

250

500

750

Biosynthesis of secondary metabolites

Energy metabolism

Global and overview maps

Glycan biosynthesis and metabolism

Metabolism of terpenoids and polyketides

Lipid metabolism

Metabolism of other amino acids

Nucleotide metabolism

Xenobiotics biodegradation and metabolism

Metabolism of cofactors and vitamins

Carbohydrate metabolism

Amino acid metabolism

Replication and repair

Translation

Folding, sorting and degradation

Transcription

Membrane transport

Signal transduction

Cellular community - prokaryotes

Cell mobility

Cell growth and death

Transport and catabolism

Pathway categories

1000

Cellular community - eukaryotes

Number of genes

**B**

RNA processing and modification

Chromatin structure and dynamics

Energy production and conversion

Cell cycle control, cell division, chromosome partitioning

Amino acid transport and metabolism

Nucleotide transport and metabolism

Carbohydrate transport and metabolism

Coenzyme transport and metabolism

Lipid transport and metabolism

Translation, ribosomal structure and biogenesis

Transcription

Replication, recombination and repair

Cell wall/membrane/envelope biogenesis

Cell mobility

Posttranslational modification, protein turnover, chaperones

Inorganic ion transport and metabolism

Secondary metabolites biosynthesis, transport and catabolism

Function unknown

General function prediction only

A:

B:

C:

D:

E:

F:

G:

H:

I:

J:

K:

L:

M:

N:

O:

P:

Q:

R:

S:

Signal transduction mechanisms

Intracellular trafficking, secretion and vesicular transport

T:

U:

Defence mechanisms

**KOG Function Classification**

Number of genes

Extracellular structure

V:

W:

A

B

C

D

E

F

G

H

I

J

K

L

M

N

O

P

Q

R

S

T

U

V

W

Nuclear structure

Cytoskeleton

X:

Y:

Function class

X

Y

0

200

400

600

800

1000

Genetic Information Processing

Environmental Information Processing

Cellular Process

Metabolism


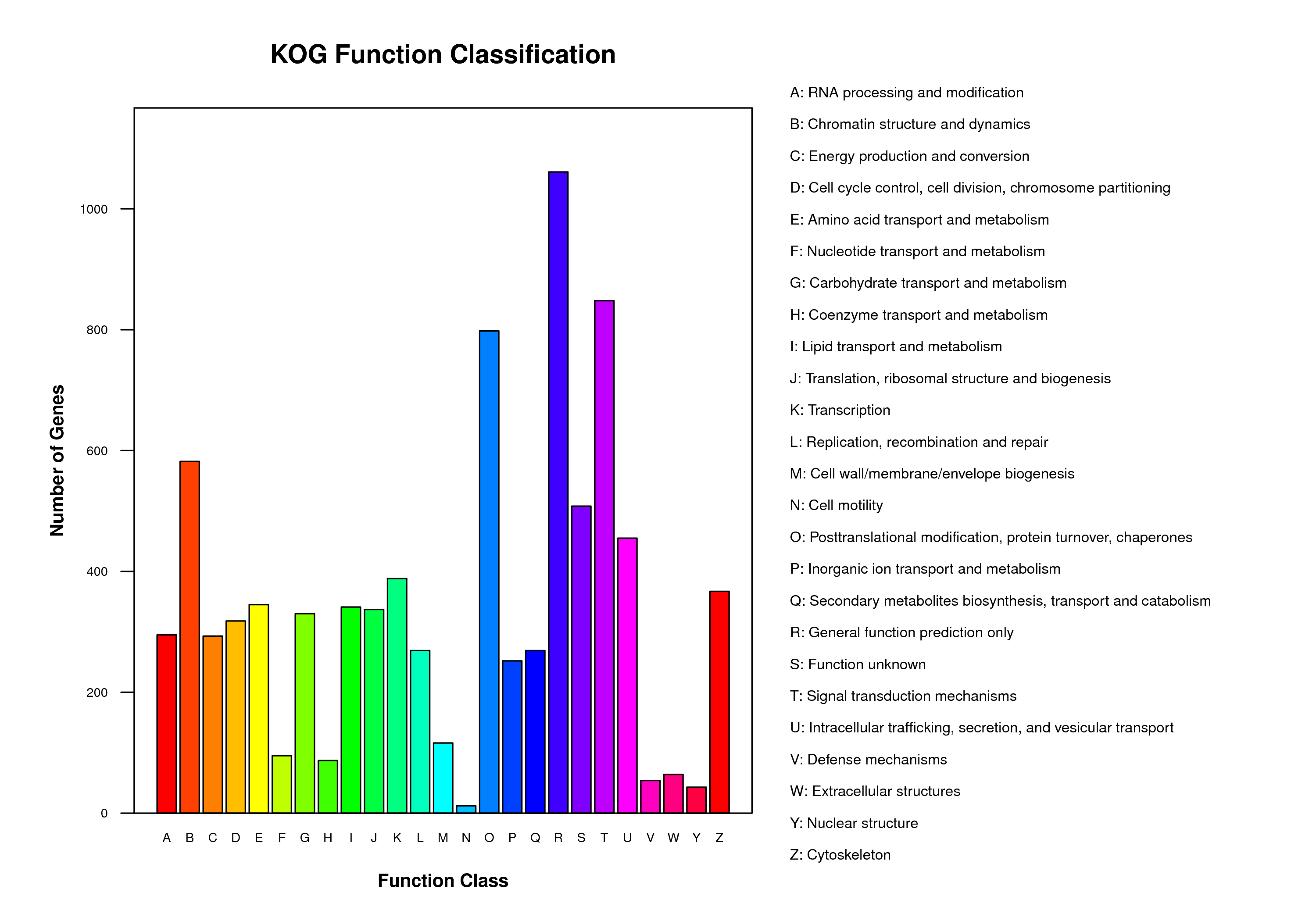


**Supplementary Figure 2.** **KEGG and Eukaryotic clusters of orthologous groups (KOG) functional annotation**. **(A)** Functional classification of KEGG pathways for assembled genes. The KEGG pathways were summarised in four main categories: blue, Metabolism; red, Cellular Processes; gold, Environmental Information Processing and green, Genetic Information Processing. The y-axis indicates the category of the metabolic pathways. The x-axis indicates the number of genes annotated under that pathway in the total number of annotated genes. **(B)** KOG classification of assembled genes. Functional predictions of genes were classified into 25 functional classes according to the KOG database.

AA: Auxiliary Activities

CBM: Carbohydrate-Binding Modules

CE: Carbohydrate Esterase's

GH: Glycoside Hydrolases

GT: Glycosyl Transferases

PL: Polysaccharide lyases


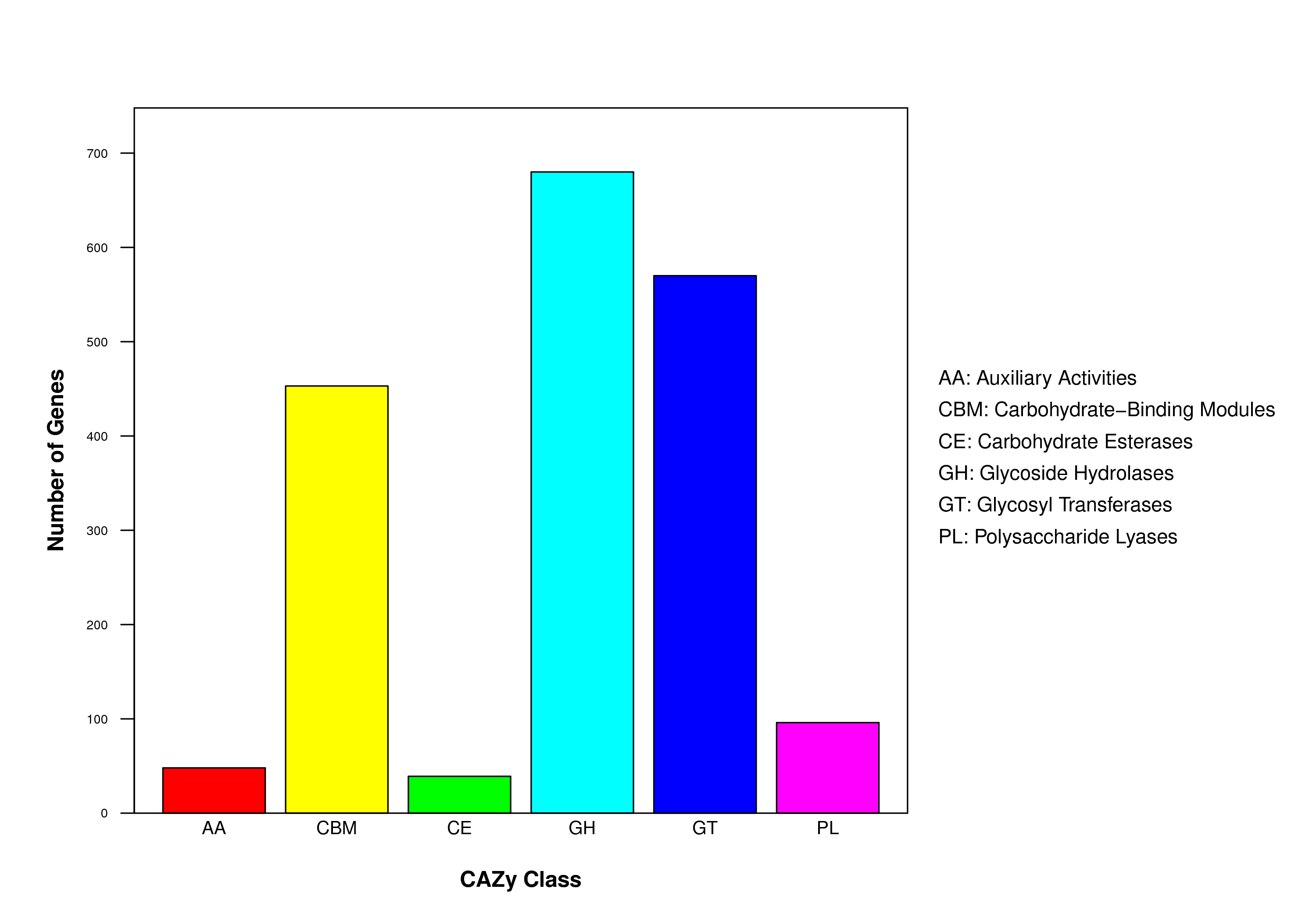


Number of genes

Function class

AA

CBM

CE

GT

GH

PL

0

100

200

300

400

500

600

700

**Supplementary Figure 3.** **Carbohydrate active enzyme (CAZy) classes of assembled genes**. The x-axis indicates the category of the CAZy class. The y-axis indicates the number of genes annotated for each the CAZy classes.


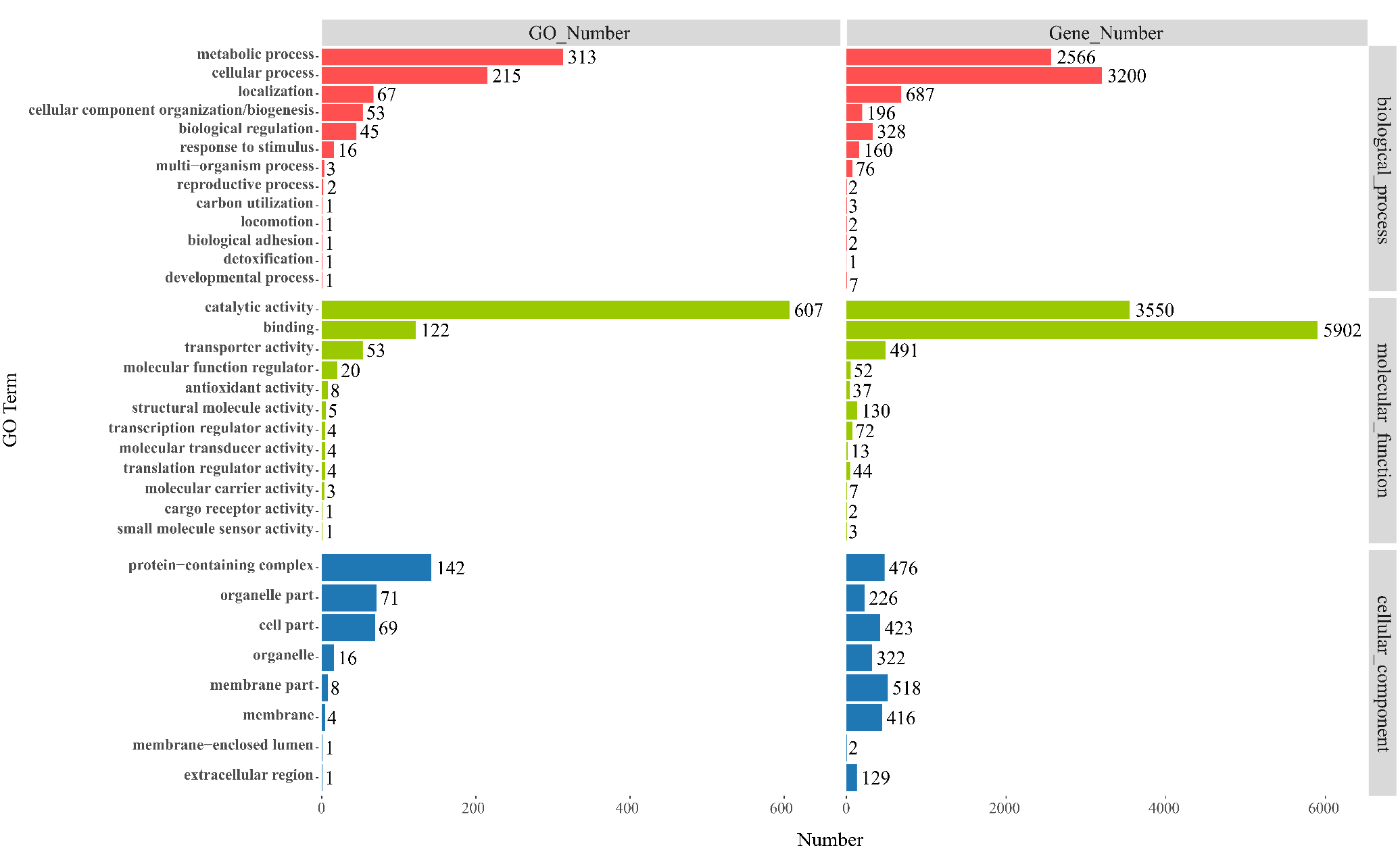


**Supplementary Figure 4.** **Gene Ontology enrichment and gene distribution of assembled genes**. The GO terms were summarised into three main categories: biological process, Molecular function, and cellular component. The y-axis indicates the GO terms within each category. The x-axis indicates the number of GO IDs and genes associated with each GO term.


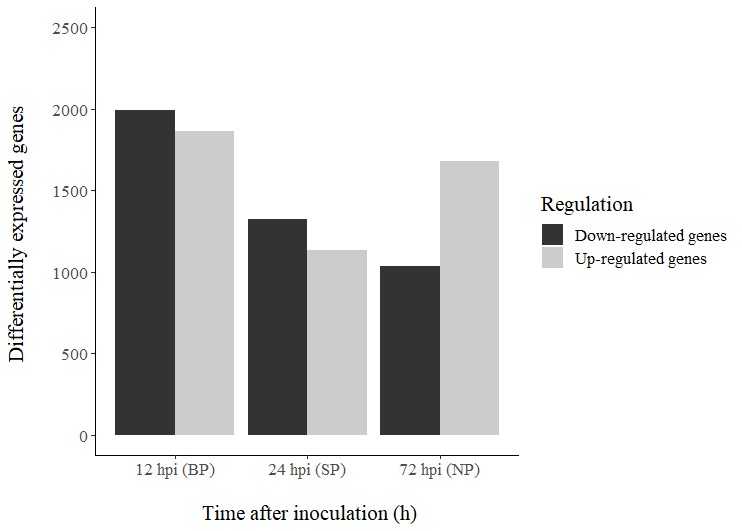

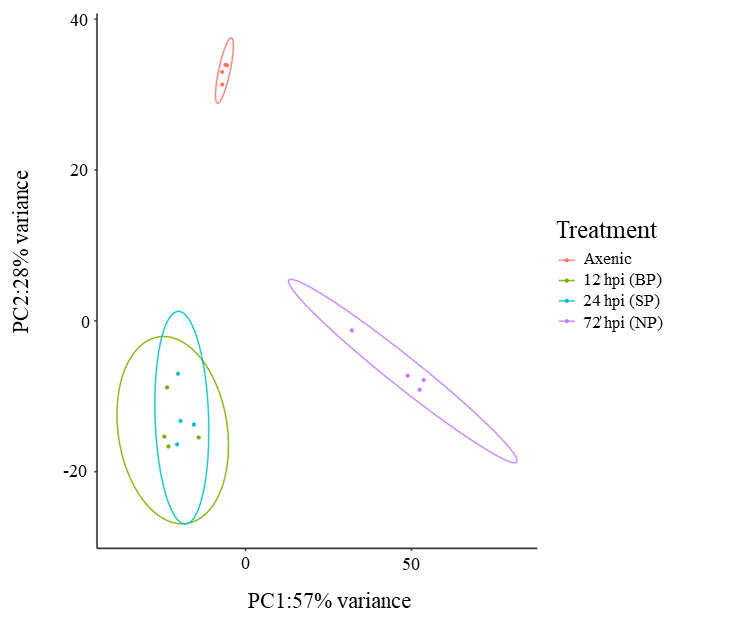

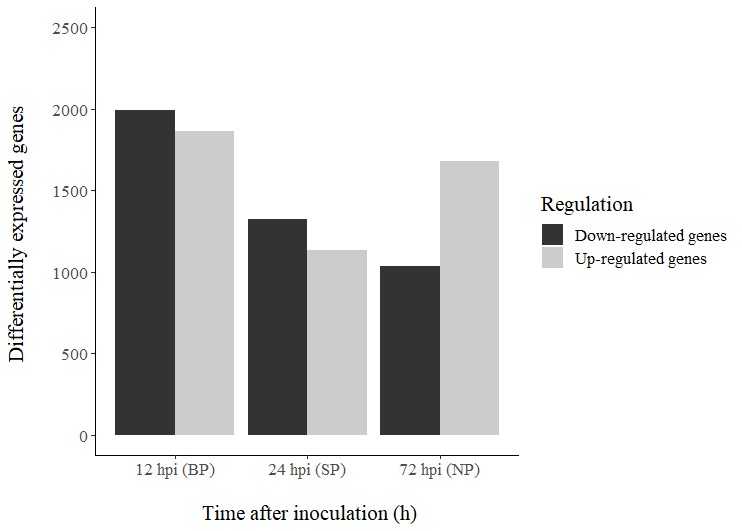

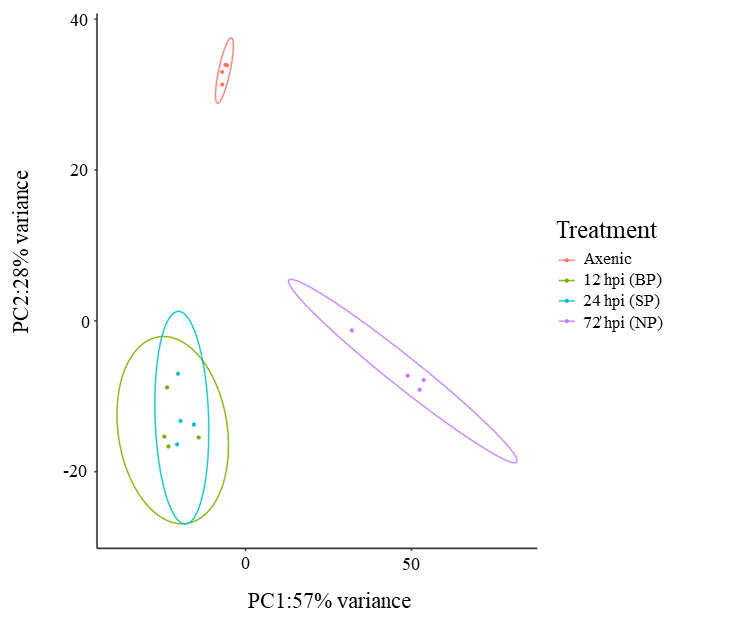

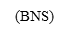


**B**

**A**

0

20

40

-20

0

50

Log2FC


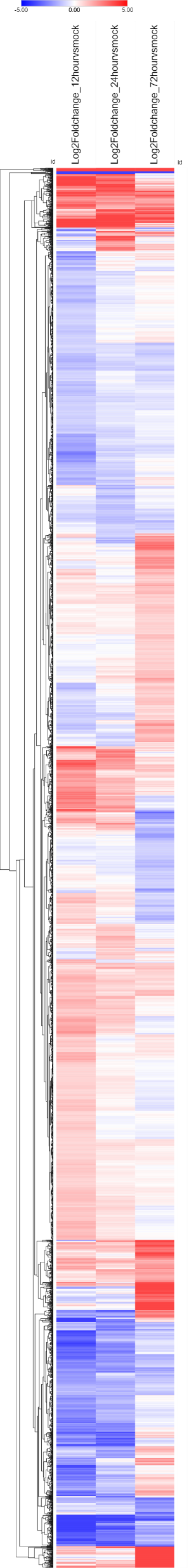


-5

5

0

72 hpi (NP)

24 hpi (BNS)

12 hpi (BP)

Axenic

**C**


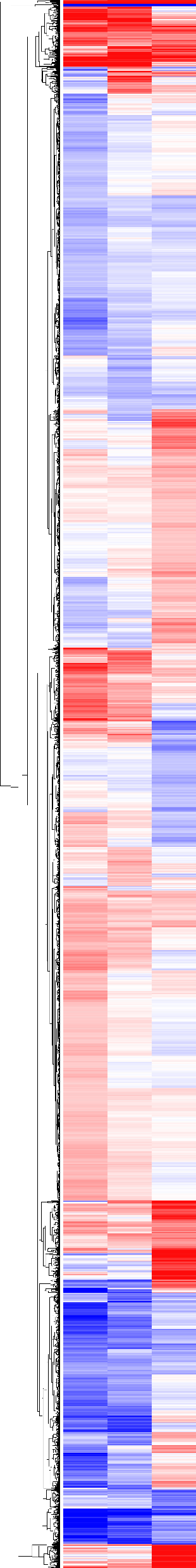


12 hpi (BP)

24 hpi (BNS)

72 hpi (NP)

12 hpi (BP)

24 hpi (BNS)

72 hpi (NP)

Time after inoculation (h)

Differentially expressed genes

0

500

1000

1500

2000

2500

Regulation

Down-regulated genes

Up-regulated genes

PC1: 57% variance

PC2: 28% variance

Treatment

**Supplementary Figure 5. Genome-wide evaluation of RNA-sequencing data in *Phytophthora medicaginis* colonising chickpea var. ‘Sonali’ roots**. **(A)** Hierarchical clustering of all *P. medicaginis* significantly differentially expressed genes in at least one timepoint (*p* < 0.05, Log_2_FC of ≥ 1 and ≤ -1)*.* **(B)** Absolute number of significantly up-regulated and down-regulated genes in *P. medicaginis* at 12, 24 and 72 hpi (*padj* < 0.05, Log_2_FC > 1 and < -1). **(C)** Principal component analysis of RNA-seq samples at 12-, 24- and 72-hours post inoculation (hpi), and axenic control.

**A**

**B**


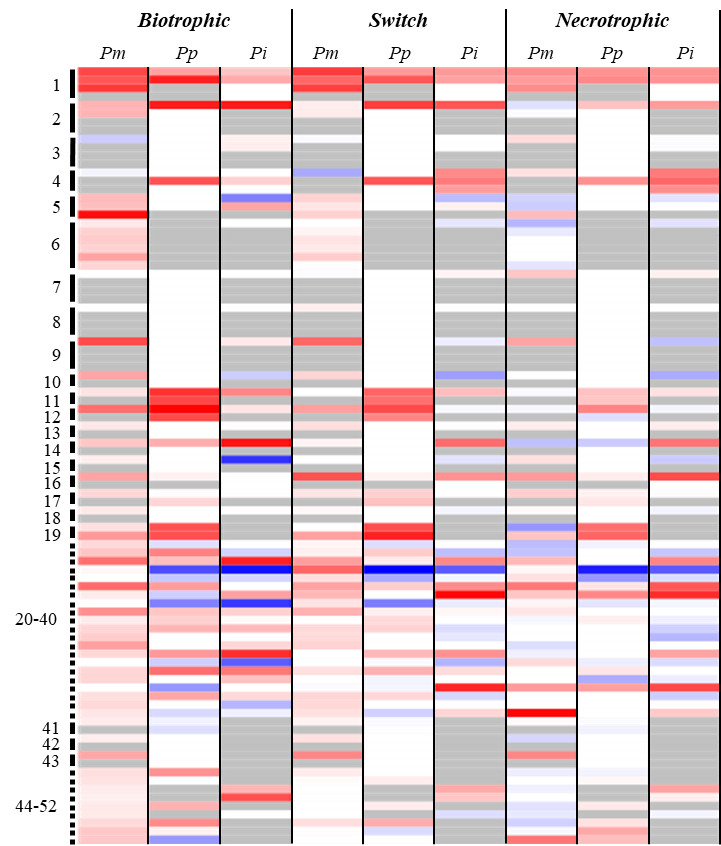


*Pectate lyase*

*Peptidase*

*Hypothetical protein*

*LysM domain containing protein*

*Hypothetical protein*

*Glycoside hydrolase*

*Splicing factor 3b, subunit 1*

*Cellulose-binding domain protein*

*Carbonic anhydrase*

Log2FC


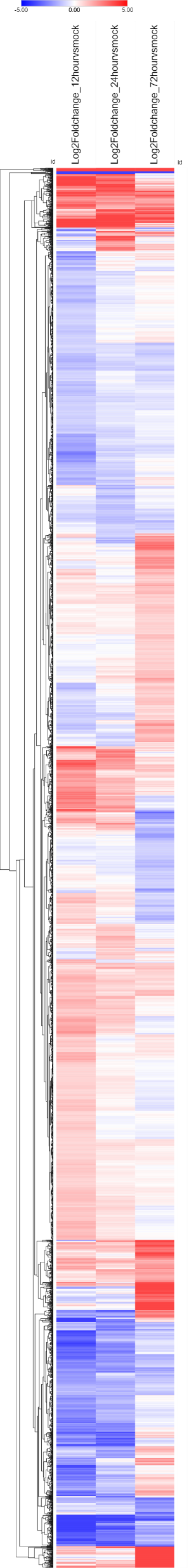


-9

9

0

**Biotrophic**

**BNS**

**Necrotrophic**


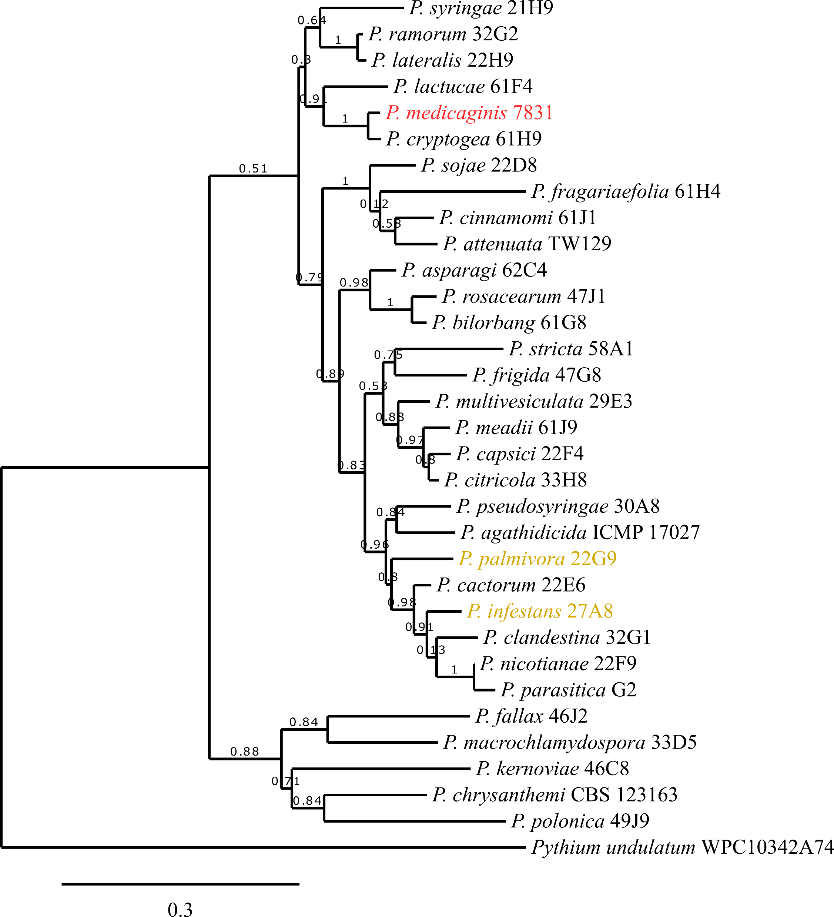


**Supplementary Figure 6. Putative *Phytophthora medicaginis* small secreted proteins (SSPs) display similar expression patterns with orthologs in distantly related *Phytophthora* species during three phases of hemibiotrophic infection**. **(A)** Maximum likelihood phylogenetic tree with 1000 bootstrap replicates based on Internal Transcribed Spacer (ITS) of 32 *Phytophthora* spp. encompassing all the subclades within the *Phytophthora* genus. *Pythium undulatum* was used as the outgroup to root the tree. The topology and branch lengths are shown. The *P. medicaginis* isolate 7831 used in this study is highlighted in red and two distantly related species *P. palmivora* and *P. infestans* used for orthologous analysis are shown in gold. **(B)** Heatmap of *P. medicaginis* significantly up-regulated SSPs and orthologous genes in *Phytophthora palmivora* and *Phytophthora infestans* during three phases of hemibiotrophic infection (*p* < 0.05, Log_2_FC > 1 and < -1). Grey indicates lack of this gene within a given genome. The numbers on the left hand side of the figure indicate the orthologous cluster. The three phases of infection (biotrophic, BNS and necrotrophic) are delineated at the top of the diagram. *Pm: Phytophthora medicaginis*; *Pp*: *Phytophthora palmivora*; *Pi*: *Phytophthora infestans*. Examples of genes displaying similar expression patterns among the species during the three phases are indicated next to the cluster they occur in on the left.

## Supplementary Tables

**Supplementary Table 1. Primers and double-stranded interfering RNAs used in this study**.

| **Gene ID** | **Sequence length (bp)** | **Primer name** | **Primer sequence (5'-3')** |
| --- | --- | --- | --- |
| *ITS* | 809 | DC6 (forward) | GAGGGACTTTTGGGTAATCA |
|  |  | ITS4 (reverse) | TCCTCCGCTTATTGATATGC |
| *Phytmed_10271* | 411 | 10271_dsiRNA_1 | CAGTTCTTCTACCGGTCCTTTCAGAAT |
|  |  | 10271_dsiRNA_2 | TGTTCATGGAACCCTAGATATTGCCGA |
| Scrambled dsiRNA | NA | Scrambled | CUUCCUCUCUUUCUCUCCCUUG |

**Supplementary Table 2. Predicted RxLR proteins in the genome of *Phytophthora medicaginis***.

**Supplementary Table 3. Predicted CRN proteins in the genome of *Phytophthora medicaginis***.

**Supplementary Table 4. Predicted NLP proteins in the genome of *Phytophthora medicaginis***.

**Supplementary Table 5. Significantly differentially expressed *Phytophthora medicaginis* genes at 12, 24 and 72 hours post inoculation in chickpea roots (*p* < 0.05, -1 > Log2FC > 1)**.

**Supplementary Table 6. Gene ontology enrichment analysis of the combined and separated uniquely upregulated and downregulated *Phytophthora medicaginis* genes at 12 (biotrophic phase), 24 (biotrophic to necrotrophic switch (BNS) phase), 72 (necrotrophic phase) hours post inoculation in chickpea roots**.

**Supplementary Table 7. *Phytophthora medicaginis* small secreted proteins regulated at three time points post inoculation in chickpea roots**.

**Supplementary Table 8. Gene Ontology enrichment analysis of host genes co-expressed with *Phytmed_10271*, *Phytmed_14744*, *Phytmed_17143*, *Phytmed_3407*, *Phytmed_8330*, *Phytmed_8971*, and *Phytmed_601***.

**Supplementary Table 9. BLAST results for *Phytmed_10271* amino acid sequence**.

**Supplementary Table 10. Chickpea genes co-expressed with *Phytophthora medicaginis* small secreted protein *Phytmed_10271****.*

**Supplementary Table 11. Significantly differentially regulated chickpea genes in *Phytmed_10271* knockdown (p < 0.05, -1 > Log2FC > 1)**.

## Supplementary Methods

**Supplementary Method 1. Phylogenetic analysis of *Phytophthora medicaginis* isolate 7831**.

The Internal Transcribed Spacer (ITS) sequences of 31 *Phytophthora* species encompassing all the (sub)clades within the molecular phylogenetic concatenated-sequence tree (TreeBASE S22998) were downloaded from Genbank (Benson et al., 2018). *Pythium ultimatum* was used as the outgroup and the ITS sequence for this species was downloaded from Genbank. For *P. medicaginis* isolate 7831, we amplified and sequenced the ITS fragment using the forward and reverse primers, DC6 and ITS4, respectively (Supplementary Table S1). The conditions for PCR were 96 °C 2 min; 10 cycles of 95 °C for 30 s, 54 °C for 30 s and 72 °C for 1 min; 25 cycles of 95 °C for 30 s, 56 °C for 30 s and 72 °C for 1 min; and a final round of 72 °C for 7 min. The PCR fragment was sequenced by Sanger sequencing at Western Sydney University. A maximum-likelihood phylogenetic tree was generated using the webtool phylogeny.fr (http://phylogeny.fr/) with default parameters (Dereeper et al., 2008).

**Supplementary Method 2. Orthologous analysis of *Phytophthora medicaginis* SSPs in distantly related species**.

For orthologous analysis of *P. medicaginis* differentially expressed SSPs, we identified 161 SSPs that were significantly up-regulated in at least one of the timepoints (12, 24 and 72 hpi) encompassing the three phases of *P. medicaginis* infection and used these to identify orthologs in two more distantly related *Phytophthora* species: *P. palmivora* and *P. infestans*. The *P. palmivora* proteome (Evangelisti *et al.*, 2017) and the *P. infestans* proteome were used for these analyses. The 161 *P. medicaginis* SSPs were concatenated into one Fasta file with the proteomes of *P. palmivora* and *P. infestans* to generate a local blast database using makeblastdb, after which an all vs all homology search was performed using blastp (Camacho *et al.*, 2009). The blast output was used as input to perform clustering of orthologous genes using FastOrtho (ttp://enews. patricbrc.org/) with 70% cut-off for both percentage identity and percent match to identify orthologs with high sequence similarity. The single and multiple copy gene orthologs were then used for expression analysis at the same key stages of infection including biotrophic phase, BNS phase and necrotrophic phase between *P. medicaginis*, *P. palmivora* and *P. infestans*.

**Supplementary Method 3. RNA-seq analysis of orthologous genes during three phases of hemibiotrophic infection**.

We identified 25 publications investigating gene expression patterns across phases of infection in different hemibiotrophic pathogens. We then filtered this list to identify publications considering *Phytophthora* and the BNS phase (Evangelisti *et al.*, 2017, Ma *et* al., 2018, Rodenburg *et al.*, 2019). Of these, we selected the study by Evangelisti *et al.* (2017), because the study focused on a root pathogen *P. palmivora*, like our study and RNA-seq data was publicly available. Although the study by Rodenburg *et al.* (2019), focused on the leaf *Phytophthora* model *P. infestans*, we included *P. infestans* because it is a model and the study also generated RNA-seq data for the similar phases of infection as our study. RNA-seq data for *P. palmivora* at specific timepoints relating to the three key stages of hemibiotrophic infection, namely biotrophic (6-24 hpi), BNS (30 hpi) and necrotrophic (48-72 hpi) in tobacco (*Nicotiana benthamiana*) roots, and axenic were downloaded from the European Bioinformatics Institute (EBI; https://www.ebi.ac.uk/) under sequence read archive SRP096022 (Evangelisti *et al.*, 2017). Similarly, RNA-seq data for *P. infestans* encompassing the same three infection stages including biotrophic (48-72hpi), BNS (72hpi) and necrotrophic (96-120 hpi) in tomato (*Solanum lycopersicum*) leaves, and axenic were downloaded from EBI under Bioproject PRJNA516028 and PRJNA361417, respectively (Rodenburg *et al.*, 2019). The RNA-seq reads were trimmed to remove low-quality sequences and adapters with default parameters using CLC Genomics Workbench 12 (Qiagen, Denmark). *P. palmivora* reads were re-aligned to the *P. palmivora* de novo transcriptome CDS (Evangelisti *et al.*, 2017), while *P. infestans* reads were re-aligned to the primary transcripts of *P. infestans* genome using CLC Genomics Workbench 12 (Haas *et al.*, 2009). To be most comparable to our analyses with *P. medicaginis,* we performed mapping using the same parameters as n this study. Statistically significant transcripts with an FDR of *p* < 0.05 and Log_2_FC > 1 and < -1 compared to axenic control were kept for further analysis. A heatmap of the single and multiple copy orthologous gene clusters between *P. medicaginis*, *P. palmivora* and *P. infestans* for each phase of infection was generated with Log_2_FC values using Morpheus (<https://software.broadinstitute.org/morpheus/>).

**References**

Benson, D. A., Cavanaugh, M., Clark, K., Karsch-Mizrachi, I., Ostell, J., Pruitt, K. D., et al. (2018). GenBank. *Nucleic Acids Res.* 46, D41. doi: 10.1093/nar/gkx1094

Camacho, C., Coulouris, G., Avagyan, V., Ma, N., Papadopoulos, J., Bealer, K., et al. (2009). BLAST+: architecture and applications. *BMC Bioinf.* 10, 1–9. doi: 10.1186/1471-2105-10-421

Dereeper, A., Guignon, V., Blanc, G., Audic, S., Buffet, S., Chevenet, F., et al. (2008). Phylogeny. fr: robust phylogenetic analysis for the non-specialist. *Nucleic Acids Res.* 36, W465–W469.

Evangelisti, E., Gogleva, A., Hainaux, T., Doumane, M., Tulin, F., Quan, C., et al. (2017). Time-resolved dual transcriptomics reveal early induced *Nicotiana benthamiana* root genes and conserved infection-promoting *Phytophthora palmivora* effectors. *BMC Biol.* 15, 1–24. doi: 10.1186/s12915-017-0379-1.

Haas, B. J., Kamoun, S., Zody, M. C., Jiang, R. H., Handsaker, R. E., Cano, L. M., et al. (2009). Genome sequence and analysis of the Irish potato famine pathogen *Phytophthora infestans*. *Nature* 461, 393–398. doi: 10.1038/nature08358

Ma, H., Shen, D., Wu, Y., Xu, H., and Dou, D. (2018). RNA-seq for comparative transcript profiling of *Phytophthora capsici* during its interaction with *Arabidopsis thaliana*. *Physiol. Mol. Plant Pathol*. 102, 193–199. doi: 10.1016/j.pmpp.2018.03.005

Rodenburg, S. Y., Seidl, M. F., Judelson, H. S., Vu, A. L., Govers, F., and de Ridder, D. (2019). Metabolic model of the *Phytophthora infestans*-tomato interaction reveals metabolic switches during host colonization. *MBio* 10, e00454–e00419. doi: 10.1128/ mBio.00454-19.
