# Supplementary material for: Functional genomics identifies a small secreted protein that plays a role during the biotrophic to necrotrophic shift in the root rot pathogen Phytophthora medicaginis
Source: Front Plant Sci. 2024 Aug 19;15:1439020. doi: 10.3389/fpls.2024.1439020 (PMC11366588; doi:10.3389/fpls.2024.1439020)
Supplement: Supplementary file 12 [file DataSheet2.pdf]

#File S1. R script used to predict RxLR: arginine-any amino acid-leucine-arginine effector, CRN: Crinkler and Necrosis, NLP: Nep1-like protein genes in the genome of *Phytophthora medicaginis* isolate 7831.

```
#EffectR script (Tabima and Grünwald, 2019) using custom motif search

#Load packages

install.packages("seqinr")

install.packages("ggplot2")

install.packages("effectR")


#Pmed proteome

library(effectR)

pkg <- "effectR"

fasta.file <- system.file("extdata", "Phytmed.aa.fa", package = pkg)



library(seqinr)

ORF <- read.fasta(fasta.file)

head(ORF, n = 2)



#REGEX search

REGEX_RxLR <- regex.search(sequence = ORF, motif = "RxLR")

#RxLR.cand <- regex.search(seq=ORF, motif = "custom", reg.pat =
"^\\w{10,40}\\w{1,96}R\\wLR\\w{1,40}[ED][ED][RK]")


REGEX_CRN <- regex.search(sequence = ORF, motif = "CRN")

#crn.cand <- regex.search(seq=ORF, motif = "custom", reg.pat = "^\\w{1,90}LFLAK\\w+")


nlp.cand <- regex.search(seq=ORF, motif = "custom", reg.pat = "GHRHDWE")


length(crn.cand)

head(crn.cand)
```

```
#Hmm step
candidate.rxlr <- hmm.search(original.seq = fasta.file, regex.seq = REGEX_RxLR,
                             mafft.path = "", num.threads = 2,
                             hmm.path = "")

candidate.crn <- hmm.search(original.seq = fasta.file, regex.seq = REGEX_CRN,
                             mafft.path = "", num.threads = 2,
                             hmm.path = "")

candidate.nlp <- hmm.search(original.seq = fasta.file, regex.seq = nlp.cand,
                            mafft.path = "", num.threads = 2,
                            hmm.path = "")

#REGEX results
head(candidate.rxlr$RxLR.cand, n = 2)
head(candidate.crn$crn.cand, n = 2)

#HMMER results
head(candidate.rxlr$HMM, n = 2)
head(candidate.crn$HMM, n = 2)

#HMM profile
head(candidate.rxlr$HMM_Table)
head(candidate.crn$HMM_Table)

#Obtaining non-redundant effectors and motif summaries
summary.list <- effector.summary(candidate.rxlr, motif='RxLR')
summary.list <- effector.summary(candidate.crn, motif='CRN')
summary.list <- effector.summary(candidate.nlp, motif='custom', reg.pat="GHRHDWE")
#summary.list <- effector.summary(candidate.crn, motif='custom', reg.pat="^\\w{1,90}LFLAK\\w+")
#summary.list <- effector.summary(crn.cand, motif='custom', reg.pat="^\\w{1,90}LFLAK\\w+")
```

```
#Motif table

write.csv(summary.list$motif.table, "RxLR_motif_table_HMM.csv")

write.csv(summary.list$motif.table, "CRN_motif_table_HMM.csv")

write.csv(summary.list$motif.table, "NLP_custom motif_table_HMM.csv")


#Non-redundant sequences

head(summary.list$motif.table, n = 2)

length(summary.list$consensus.sequences)


#Exporting the non-redundant effector candidates

write.fasta(sequences = getSequence(summary.list$consensus.sequences), names = getName(summary.list$consensus.sequences), file.out = "NLP_candidates.fasta")


#Visualizing the HMM profile using a sequence logo-like plot

hmm.logo(hmm.table = candidate.nlp$HMM_Table)
```
